# QTL mapping and candidate genes for resistance to *Fusarium* ear rot and fumonisin contamination in maize

**DOI:** 10.1186/s12870-017-0970-1

**Published:** 2017-01-21

**Authors:** Valentina Maschietto, Cinzia Colombi, Raul Pirona, Giorgio Pea, Francesco Strozzi, Adriano Marocco, Laura Rossini, Alessandra Lanubile

**Affiliations:** 1 Department of Sustainable Crop Production, Università Cattolica del Sacro Cuore, Via Emilia Parmense 84, 29122 Piacenza, Italy; 2 Parco Tecnologico Padano, Via Einstein, Loc. Cascina Codazza, 26900 Lodi, Italy; 3 Institute of Agricultural Biology and Biotechnology, CNR, Via Bassini 15, 20133 Milano, Italy; 4 Department of Agricultural and Environmental Sciences Production, Landscape, Agroenergy, Università degli Studi di Milano, Via Celoria 2, 20133 Milano, Italy

**Keywords:** *Zea mays*, *Fusarium verticillioides*, FB1 contamination, Genotyping-by-Sequencing

## Abstract

**Background:**

*Fusarium verticillioides* is a common maize pathogen causing ear rot (FER) and contamination of the grains with the fumonisin B1 (FB1) mycotoxin. Resistance to FER and FB1 contamination are quantitative traits, affected by environmental conditions, and completely resistant maize genotypes to the pathogen are so far unknown. In order to uncover genomic regions associated to reduced FER and FB1 contamination and identify molecular markers for assisted selection, an F_2:3_ population of 188 progenies was developed crossing CO441 (resistant) and CO354 (susceptible) genotypes. FER severity and FB1 contamination content were evaluated over 2 years and sowing dates (early and late) in ears artificially inoculated with *F. verticillioides* by the use of either side-needle or toothpick inoculation techniques.

**Results:**

Weather conditions significantly changed in the two phenotyping seasons and FER and FB1 content distribution significantly differed in the F_3_ progenies according to the year and the sowing time. Significant positive correlations (*P <* 0.01) were detected between FER and FB1 contamination, ranging from 0.72 to 0.81. A low positive correlation was determined between FB1 contamination and silking time (DTS). A genetic map was generated for the cross, based on 41 microsatellite markers and 342 single nucleotide polymorphisms (SNPs) derived from Genotyping-by-Sequencing (GBS). QTL analyses revealed 15 QTLs for FER, 17 QTLs for FB1 contamination and nine QTLs for DTS. Eight QTLs located on linkage group (LG) 1, 2, 3, 6, 7 and 9 were in common between FER and FB1, making possible the selection of genotypes with both low disease severity and low fumonisin contamination. Moreover, five QTLs on LGs 1, 2, 4, 5 and 9 located close to previously reported QTLs for resistance to other mycotoxigenic fungi. Finally, 24 candidate genes for resistance to *F. verticillioides* are proposed combining previous transcriptomic data with QTL mapping.

**Conclusions:**

This study identified a set of QTLs and candidate genes that could accelerate breeding for resistance of maize lines showing reduced disease severity and low mycotoxin contamination determined by *F. verticillioides*.

**Electronic supplementary material:**

The online version of this article (doi:10.1186/s12870-017-0970-1) contains supplementary material, which is available to authorized users.

## Background

*Fusarium* ear rot (FER) is a common disease of maize (*Zea mays* L.), which reduces grain yield and quality worldwide. The fungus *Fusarium verticillioides* (Sacc.) Nirenberg is the primary causal agent of FER, particularly in Southern Europe [1, 2] and in the United States [3]. This pathogen is the major producer in the grains of fumonisin mycotoxins, including fumonisin B1 (FB1). Fumonisins were classified as probable carcinogens, because of their suspected involvement in esophageal cancer and neural tube birth defects in humans, whilst in livestock they cause equine leukoencephalomalacia, porcine pulmonary edema, poultry reduced growth and hepatic and immune disorders in cattle [1, 2]. The European Union established fumonisin content thresholds of 4,000 ppb in non-processed corn, and 1,000 ppb for corn intended for direct human consumption [4], which were frequently overcome in years favorable for the pathogen. In a 3-years study (2009–2011), fumonisin contamination was detected in 90% of Southern European corn samples, with an average level of 2,200 ppb and a maximum level greater than 11,000 ppb [5].

Agronomic practices for fumonisin content reduction are ineffective when conditions for fungal growth are optimal [6]. Therefore, breeding for resistance to fumonisin contamination emerged as the most economic and environmentally safe strategy [7], and many studies focused on the search for resistance [8–13]. These studies demonstrated that genetic variation for resistance to FER and fumonisin contamination exists among inbred lines and hybrids, but there is no evidence of complete resistance to the pathogen. Despite moderate phenotypic correlations (*r* = 0.40–0.64), genotypic correlations between the two traits were high (*r* = 0.87–0.96), confirming that selection against ear rot implies the choice of genotypes with lower fumonisin contamination [14].

Quantitative Trait Locus (QTL) mapping studies in maize indicated that *Fusarium* resistance and fumonisin contamination are quantitative traits determined by small effect polygenes [15–18]. Perez-Brito and colleagues [15] identified 16 QTLs for FER resistance in two F_2:3_ populations sharing the same susceptible parent, explaining in total 11–44% of the phenotypic variation, but only three QTLs were consistent across populations. Robertson-Hoyt and coworkers [16] identified higher effect QTLs, explaining in total 31 and 47% of the phenotypic variation for FER resistance and 67 and 81% for fumonisin concentration in two independent segregating populations, respectively. These QTLs were partially consistent across populations and mapped in similar positions for both traits [16]. Heritability was estimated in the range 0.47–0.80 for FER resistance and 0.75–0.86 for fumonisin contamination depending on the population [14]. Ding and colleagues [17] carried out QTL mapping of FER resistance on a recombinant inbred line (RIL) population in different environments, detecting significant epistatic effects on FER and interactions between mapped loci and environments. Recently, a QTL for FER resistance affecting around 18% of the phenotypic variation was discovered on chromosome 4 and introgressed into Near Isogenic Lines, accounting for up to 35% of the phenotypic effect when in homozygosity [18].

The complex genetic bases of these traits and the strong influence of environmental factors hinder accurate QTL localization and effect estimates, therefore reducing the efficiency of marker assisted selection (MAS) [16]. Such limitations may be overcome by increasing population size and the number of markers used, improving ear rot phenotyping protocols and integrating data from multiple environments [19]. In particular previous QTL mapping studies on these traits were based on maps containing few hundreds restriction fragment length polymorphisms (RFLP; [15]) and single sequence repeat (SSR) markers [16–18]. In recent years, Single Nucleotide Polymorphisms (SNPs) have become the preferred genotyping system for genetic studies being the cheapest and the most abundant markers in a genome [20], e.g., 1 SNP/100 bp in maize [21]. With the advent of the Next Generation Sequencing technologies, SNP markers have shown their full potentiality with novel approaches combing SNP discovery and genotyping. For example, Elshire and coworkers [22] have developed a simple technique, called Genotyping-by-Sequencing (GBS), in which multiplexed libraries based on the reduction of genome complexity through restriction with enzymes are constructed to preferentially target sequences in low copy genomic regions, minimizing reads in repetitive regions that are frequent in maize [23]. GBS has been applied for population studies, germplasm characterization, breeding and trait mapping in a number of plant species, including maize, barley, wheat, soybean, switchgrass and rice [24–28]. Two recent genome-wide association studies were performed in maize to detect allele variants associated with increased resistance to FER, resulting in three SNPs with significant effects on chromosome 1, 5 and 9 [29] and seven SNPs on chromosomes 4, 5 and 9 [30].

The aim of this work was the mapping of QTLs and identification of candidate genes for FER resistance and reduced FB1 contamination in a F_2:3_ progeny, derived from the cross between a resistant (CO441) and a susceptible (CO354) commercial maize line previously used for molecular characterization of response to *Fusarium* [31–35]. Phenotypic evaluation in two different sowing times for two consecutive years was carried out in order to take into account the variation due to environmental effects. Among the multitude of published inoculation methods, the toothpick (inoculation with mycelium) and the side-needle techniques (inoculation with conidia) were chosen to phenotype the population, since the former is known for its greater aggressiveness and the latter mimics natural infection [36]. SNPs, derived by GBS, and SSR markers were used to build a linkage map as a basis for detection of QTLs for FER and FB1 contamination in maize. Finally, candidate genes for resistance to the pathogen are proposed based on integration of QTL analysis results with transcriptomic data, previously obtained on the two parents artificially inoculated with *F. verticillioides* [34].

## Results

### Disease development and weather conditions during flowering and post-inoculation periods

The F_2:3_ population (CO441xCO354) was phenotyped in 2011 and 2012 and in an early (A) and late (B) sowing date for each phenotyping year.

Weather conditions during two periods of maize development, flowering and kernel drying, are critical for fumonisin contamination of the kernels [2]. The weekly means of maximum and minimum temperatures, maximum and minimum relative humidity and rainfall occurring in the experimental field in 2011 and 2012 from flowering until harvest are reported in Additional file 1: Figure S1.

The temperatures and relative humidity differed significantly in the period between flowering and harvest of 2011 and 2012, according to Kruskal-Wallis test (*P <* 0.05). In particular, during the flowering period, significantly higher temperatures occurred in 2012A and B, with median values of maximum temperature of 32.8 and 31.6 °C, respectively, in comparison to 2011A (median = 29.6 °C) and 2011B (median = 28.4 °C). Moreover, maximum relative humidity was significantly lower in 2012, with median values of 75% (2012A), 72% (2012B), 81% (2011A) and 88% (2011B). No significant changes in minimum relative humidity and rainfall were found between the four sowing times.

In the post-inoculation period, relative humidity was significantly lower in 2012, with the maximum and minimum values of 73 and 31% (2012A), of 74 and 31% (2012B), of 87 and 36% (2011A) and of 87 and 36% (2011B), respectively. Maximum temperatures were significantly higher in 2012, with median values higher than 32 °C, whilst in 2011 they were lower than 30 °C. The minimum temperatures and rainfall did not significantly differ in the four sowing times.

The higher temperatures and the lower humidity of 2012 affected disease development, since the population mean in 2011 ranged from 3.3 to 3.6 for FER severity and from 45 to 60 ppm for FB1 contamination, and higher mean values were reached in 2012 for both traits (3.2–4.2 and 37–68 ppm, respectively).

### Phenotypic analysis for FER severity and FB1 contamination

**Fig. 1:**
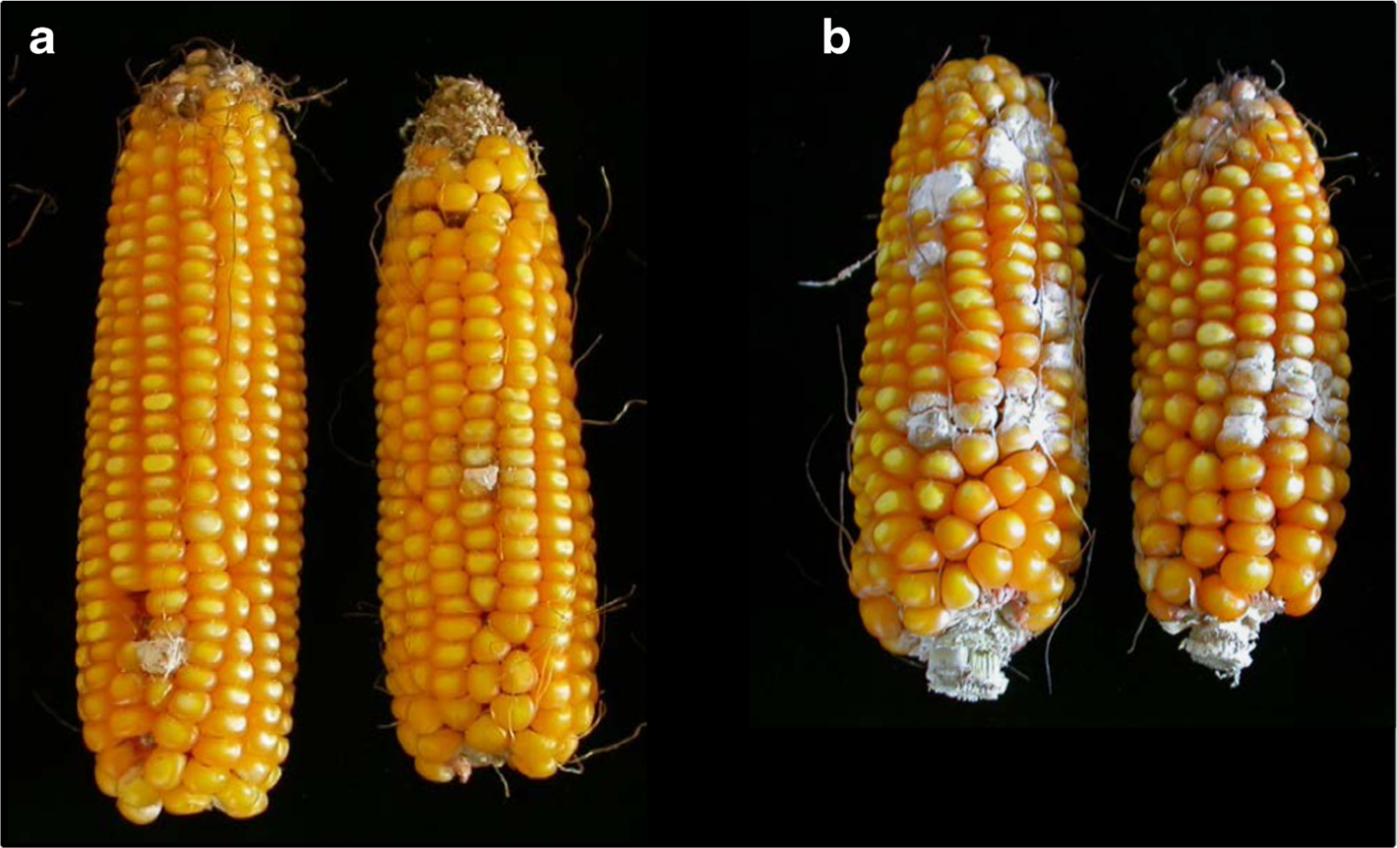
Phenotypic variation in *Fusarium* ear rot severity at harvest among the parental lines in artificially inoculated ears with *F. verticillioides*. The resistant CO441 is represented by the two ears on the left (**a**) and the susceptible CO354 by the two ears on the right (**b**)

The CO441 and CO354 parents and the 188 F_3_ progenies were visually scored for FER severity and the phenotypic variation between parents is shown in Fig. 1. Ears infected with either the toothpick (T) or the side-needle (F) method in early (A) and late sowing (B) of 2011 and 2012 were then pooled and FB1 content was estimated by Near-Infrared Spectroscopy (NIRS).

**Fig. 2:**
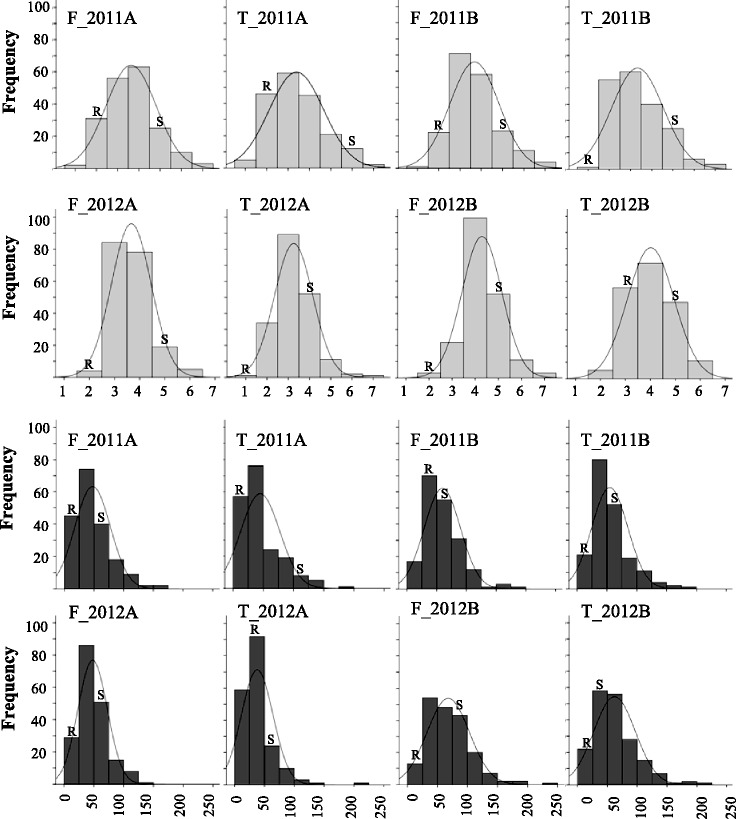
Distributions of F_3_ progenies for *Fusarium* ear rot severity (*light grey*) and fumonisin B1 contamination (*dark grey*) in early (A) and late (B) sowings in 2011 and 2012 with the side-needle (F) and toothpick (T) inoculation methods and normal distribution curve. The classes related to CO441 and CO354 parent values are indicated with R (resistant) and S (susceptible), respectively. X-axis for FER severity represents the 1–7 classes of infection of the ear. X-axis for FB1 contamination represents the mycotoxin content measured by NIR spectroscopy and expressed in ppm

The distributions of FER and FB1 traits in the F_3_ population are shown in Fig. 2. The Shapiro-Wilk test showed that none of the traits were normally distributed (*P <* 0.01) and they exhibited positive skewness with a leptokurtic pattern in most cases (Fig. 2). Positive skewness for FER scores was due to the high frequencies of 3^rd^ and 4^th^ classes (4–10 and 11–25% of infection on the ear, respectively), and the high frequency of low-contaminated samples (<50 ppm) for FB1 contamination. Transgressive segregation was observed for both FER and FB1 in both years of analysis and sowing times (Fig. 2): several families showed higher and lower levels of FER severity and FB1 contamination compared to the parents and some were consistent across years, inoculation methods and sowing times (data not shown).

A summary of FER scores and FB1 contents observed in the 2 years for F_3_ families and parents is shown in Additional file 2: Table S1. The CO441 parent showed in all conditions lower disease severity and fumonisin content (FER and FB1 mean values of 1.0–2.8 and of 7–40 ppm, respectively) compared to the CO354 parent (FER and FB1 mean values of 4.0–6.4 and of 47–116 ppm, respectively). The mean of the two traits in the population was always located between the values of the two parents, except for FB1 T in 2012 A and B. Friedman test revealed significant (*P <* 0.001) differences among groups determined by the inoculation technique, sowing time and year for both FER and FB1 traits (Additional file 2: Table S1). In particular, the medians for FER of the population inoculated with the toothpick appeared significantly (*P <* 0.05) lower compared to the side-needle method, according to Wilkoxon signed-rank test with Bonferroni correction. No significant differences in the FER mean-ranks were detected in 2011A, 2011B and 2012A, whilst the highest infection levels were associated to 2012B. In contrast, Wilkoxon signed-rank test results for FB1 contamination indicated that the two different inoculation techniques did not significantly affect fumonisin content in 2011, but in 2012 the side-needle inoculation produced a lower level of contamination in both sowing times. In agreement with results for FER, 2012B samples showed significantly higher contamination (Additional file 2: Table S1).

Pearson’s correlation coefficients between phenotypic traits, inoculation methods, sowing dates and years are shown in Table 1. Regarding the relations between traits within the same sowing date and same inoculation method, significant positive correlations (*P <* 0.01) were detected between FER and FB1 contamination, ranging from 0.72 (F_2012B and T_2012A) to 0.81 (T_2011B) (Table 1I). The correlation between the inoculation techniques (F and T), within the same sowing time, was more consistent for FER (*r* = 0.58–0.77, *P <* 0.01) than for FB1 contamination (*r* = 0.48–0.59) (Table 1I). Correlations in different sowing times, within the same year, were low (*r* = 0.35–0.50, *P <* 0.01) (Table 1II). In addition, correlation between different years appeared low (*r* = 0.31–0.57, *P <* 0.01), indicating a seasonal influence on the outcome of *Fusarium* infection (Table 1II).

**Table 1:** Pearson’s correlation coefficients between *Fusarium* ear rot severity (FER) and fumonisin B1 concentration (FB1) of F_3_ families, measured with two inoculation methods (F = side-needle and T = toothpick), in early (A) and late (B) sowings of 2011 and 2012. I. Correlation between traits within the same sowing time. II. Correlation between traits across different sowing times and different years

I		FB1 F	FER T	FB1 T		
FER F	2011 A	0.75**	0.73**	0.52**		
	2011 B	0.79**	0.77**	0.59**		
	2012 A	0.77**	0.62**	0.46**		
	2012 B	0.72**	0.58**	0.51**		
FB1 F	2011 A		0.58**	0.51**		
	2011 B		0.58**	0.57**		
	2012 A		0.58**	0.59**		
	2012 B		0.49**	0.48**		
FER T	2011 A			0.77**		
	2011 B			0.81**		
	2012 A			0.72**		
	2012 B			0.78**		
II	Correlation between sowings		Correlation between years			
trait	2011A-2011B	2012A-2012B	2011A-2012A	2011A-2012B	2011B-2012A	2011B-2012B
FER F	0.38**	0.35**	0.36**	0.31**	0.48**	0.44**
FER T	0.50**	0.42**	0.42**	0.33**	0.49**	0.46**
FB1 F	0.40**	0.36**	0.47**	0.33**	0.47**	0.49**
FB1 T	0.47**	0.47**	0.40**	0.32**	0.57**	0.45**

** = significant at *P* < 0.01

Beside FB1 content and FER severity, days from sowing to silking (DTS) were registered for each F_3_ family, in order to evaluate the possible correlation between resistance to *Fusarium* inoculation and earliness in flowering. DTS showed non-normal distributions (*P <* 0.01) in 2011B, 2012A and 2012B, exhibiting positive skewness with a leptokurtic pattern (data not shown). Values ranged from 65 to 78 days in 2011A, 59–75 days in 2011B, 61–75 days in 2012A and 54–70 days in 2012B (Additional file 2: Table S1). The presence of transgressive segregants was recorded only in 2011A. A statistically significant difference in DTS depending on the year and sowing date was detected (Friedman test, *P <* 0.001), with the highest value associated to 2011A (median = 70) and the lowest with 2012B (median = 60) (Additional file 2: Table S1).

The correlation between DTS and FER and FB1 accumulation traits were evaluated by Pearson’s correlation coefficients (Additional file 3: Table S2). Significant (*P <* 0.05) low positive correlations (*r* = 0.16–0.28) were found between DTS and FB1 contamination for all sowing dates, except for 2011A. Low positive correlations (*P <* 0.01) between DTS and FER were detected only in 2011B (*r* = 0.25 (F), *r* = 0.29(T).

In conclusion, phenotypic data from the two inoculation methods in four different environments showed ample segregation for FER severity and FB1 content in the CO441xCO354 population, providing an ideal basis for genetic dissection of the resistance traits.

### Genotyping, linkage map construction and QTL analyses

Screening of 369 SSR markers on the parents CO441 and CO354 resulted in identification of a set of 95 polymorphic markers, i.e., only 25.7% of screened SSRs were suitable for linkage analyses (Additional file 4: Table S3). To improve map density and QTL resolution, GBS was applied, providing on average 1,042,757 reads per sample, 717,272 and 699,275 reads for the CO441 and CO354 parents, respectively. Variant calling against the reference B73 genome yielded 16,236 sequence variants, including 13,292 SNPs and 963 INDELs, while the remaining 1,981 variations fell in the complex or multi nucleotide polymorphism (MNP) allelic variant type. Stringent selection criteria were used to select markers for final map construction taking into account the fact that genotyping data were obtained on two different generations (F_2_ individuals for SSR markers and F_2_-like pools of F_3_ individuals for GBS; see Materials and Methods section for details). This resulted in exclusion of a high proportion of markers. During map construction, a number of markers were excluded manually based on careful inspection of their map positions and comparison with the reference genome. Finally, 383 (342 SNPs and 41 SSRs) markers were included in the final linkage map covering 3168.91 cM with an average density of 8.40 cM/marker (Additional file 5: Table S4).

QTL analyses were performed using phenotypic data (FER and FB1 content) recorded over 2 years (2011 and 2012) and two sowing times (indicated as A and B) with two inoculation methods (F or T). In addition, DTS was subjected to QTL analyses in order to exclude loci influencing flowering time and consider only loci related to resistance. The LOD value thresholds obtained by permutation test varied from 3.9 to 4.3 for all considered traits (see Materials and Methods section for details), but we considered as “stable” also QTLs with LOD values close to the threshold (difference with the threshold <0.50 LOD), if mapping to the same position of another QTL determined in another year/sowing time/inoculation technique, or for another trait.

While the traits phenotyped in our work are directly related to disease impact and thus to susceptibility, the goal in breeding is to improve resistance to the pathogen. For this reason throughout the paper we will consider as beneficial those alleles that decrease FER severity and FB1 contamination. Significant associations were mapped for FER, FB1 and DTS traits in the four environments and the two inoculation methods. Fifteen, 17 and nine associations with overlapping confidence intervals (2-LOD) in at least two inoculation methods and/or two sowing times and/or 2 years, were detected for FER, FB1 contamination and DTS, respectively (Table 2; Additional file 6: Figure S2). Overlapping QTLs in the 2-LOD intervals were referred as “integrated QTLs” and indicated by the trait code followed by the LG in which the QTL was mapped and a decimal if another QTL for the same trait was mapped in the same LG. Between the 2-LOD overlapping QTLs for each trait, the highest LOD peak value and the maximum explained phenotypic variation (and the corresponding nearest cofactor marker, the LOD peak position, the additive and dominance effect) were chosen as putative value of the integrated QTL and reported in Table 2. The new confidence interval of the integrated QTLs were calculated on the extremes of the 2- and 1-LOD interval of each overlapping QTL detected (Additional file 6: Figure S2).

**Table 2:** Mapped integrated QTLs for *Fusarium* ear rot (FER), fumonisin B1 (FB1) contamination and days to silking (DTS) by multiple QTL mapping in a F_2:3_ population (CO441xCO354) artificially infected with side-needle (F) and toothpick (T) inoculation methods in early (A) and late sowing (B) of 2011 and 2012, detected in more than 1 year/sowing/inoculation technique. Co-mapping QTLs for FER, FB1 contamination and DTS are in bold

Integrated QTL name^a^	LG.bin^b^	LOD peak^c^	R^2^ (%)^d^	LOD position^e^ (cM)	Cofactor marker^f^	Additive Effect^g^	Dominance Effect^h^	mu_A^i^	mu_H^l^	mu_B^m^	QTL detection^n^
**qFER-1**	**1.05**	**9.57**	**11**	**174.986**	**1ch92268079**	**−0.32**	**0.76**	**3.642**	**4.719**	**4.278**	**1 F; 4 T**
qFER-2.1	2.00	7.96	4.5	18.472	phi96100	0.4	−0.01	4.401	3.998	3.559	2 F; 4 F; 4 T
qFER-2.2	2.03	5.5	5.6	88.274	2ch15292681	0.26	0.46	3.637	3.842	3.125	2 T; 3 F
qFER-2.3	2.04	5.67	5.5	125.698	2ch38532783	0.23	−0.29	3.845	3.325	3.324	2 T; 4 T
**qFER-2.4**	**2.05**	**9.88**	**11.6**	**165.676**	**bnlg1909**	**0.54**	**−0.42**	**4.503**	**3.540**	**3.417**	**1 F; 4 F; 4 T**
**qFER-3**	**3.05**	**8.63**	**7.5**	**297.535**	**3ch155286584**	**−0.32**	**0.39**	**3.109**	**3.820**	**3.745**	**1 F; 1 T; 2 F**
qFER-4	4.10	7.94	8.4	313.514	4ch239495017	−0.14	0.46	3.447	4.048	3.722	3 F; 4 F
qFER-5	5.02	9.15	10	68.666	5ch10243313	−0.59	−0.49	2.786	2.891	3.975	2 F; 2 T; 4 T
**qFER-6**	**6.01**	**19.47**	**13.6**	**24.910**	**6ch85961919**	**0.46**	**−0.07**	**4.458**	**3.928**	**3.542**	**2 F; 4 F**
**qFER-7**	**7.02**	**15.45**	**17.5**	**143.720**	**bnlg1164**	**−0.51**	**−0.32**	**3.521**	**3.713**	**4.545**	**1 F; 1 T; 2 T; 3 F; 3 T; 4 T**
qFER-8	8.06	5.82	8.8	173.071	8ch152286794	0.25	0.42	3.881	4.059	3.389	3 F; 3 T
**qFER-9.1**	**9.03**	**5.11**	**8.8**	**111.706**	**9ch77768666**	**−0.44**	**−0.37**	**3.084**	**3.147**	**3.957**	**1 F; 1 T**
qFER-9.2	9.05	16.16	18.7	143.050	bnlg1270	−0.76	0.26	2.619	3.644	4.142	2 F; 2 T; 3 T
**qFER-9.3**	**9.06**	**4.83**	**4.8**	**170.642**	**9ch147073999**	**−0.11**	**−0.28**	**3.470**	**3.296**	**3.699**	**3 F; 4 T**
**qFER-9.4**	**9.07**	**8.88**	**5.1**	**221.106**	**9ch152032473**	**0.09**	**0.42**	**4.091**	**4.424**	**3.909**	**1 T; 4 F**
**qFB1-1.1**	**1.05**	**6.58**	**6.1**	**166.834**	**umc2025**	**−16.07**	**10.77**	**4.585**	**7.269**	**7.799**	**4 T; 4 F**
qFB1-1.2	1.08	6.45	5	286.955	1ch248925051	−0.2	20.07	5.366	7.393	5.406	1 F; 4 F; 4 T
qFB1-1.3	1.11	5.47	4.2	361.786	1ch289346273	1.08	−17.16	5.494	3.670	5.278	3 F; 4 F
**qFB1-2**	**2.05**	**10.73**	**17.2**	**164.890**	**bnlg1909**	**18.11**	**−10.7**	**7.350**	**4.469**	**3.728**	**1 F; 2 F**
**qFB1-3**	**3.05**	**10.77**	**5.6**	**297.535**	**3ch155286584**	**−8.98**	**4.25**	**5.552**	**6.875**	**7.348**	**3 T; 4 F**
qFB1-4.1	4.01	5.61	5.1	26.277	4ch2672212	−1.83	18.31	6.008	8.022	6.375	3 T; 4 T
qFB1-4.2	4.05	12.53	4.6	107.351	4ch35659578	−7.28	−5.38	4.396	4.586	5.852	3 F; 4 T
qFB1-4.3	4.08	11.98	4.2	193.826	4ch180229652	−5.26	−3.85	4.722	4.862	5.773	3 F; 3 T
qFB1-5	5.08	19.92	7.7	270.337	5ch211812130	−9.28	−11.02	3.779	3.604	5.634	3 F; 4 T
**qFB1-6.1**	**6.01**	**7.88**	**3.9**	**24.910**	**6ch85961916**	**3.7**	**−2.59**	**6.989**	**6.361**	**6.250**	**3 F; 3 T**
qFB1-6.2	6.05	6.24	2.9	110.387	6ch141801055	5.69	−7.98	6.977	5.611	5.840	1 F; 3 T
**qFB1-7.1**	**7.02**	**6.32**	**12.8**	**126.321**	**7ch31316454**	**−13.44**	**−10.35**	**5.809**	**6.118**	**8.497**	**1 T; 2 T**
**qFB1-7.2**	**7.02**	**12.22**	**12.4**	**143.720**	**bnlg1164**	**−19.26**	**−9.18**	**4.266**	**5.274**	**8.117**	**2 F; 3 F; 4 T**
qFB1-9.1	9.01	17.75	5.4	17.000	umc1867	9.76	−8.94	5.682	3.812	3.730	3 F; 3 T
**qFB1-9.2**	**9.04**	**8.31**	**4**	**119.706**	**9ch120504202**	**3.47**	**−11.16**	**6.472**	**5.009**	**5.779**	**3 T; 4 F**
**qFB1-9.3**	**9.06**	**10.86**	**5.5**	**180.101**	**9ch147547533**	**−10.39**	**−6.63**	**5.369**	**5.745**	**7.448**	**3 T; 4 F**
**qFB1-9.4**	**9.07**	**11.28**	**5.5**	**212.419**	**9ch154004726**	**−11.12**	**−8.29**	**5.296**	**5.579**	**7.521**	**1 T; 2 T; 3 T**
qDTS-1.1	1.01	13.8	9.9	28.341	1ch4761508	1.04	−0.55	6.393	6.234	6.185	2; 4
qDTS-1.2	1.06	17.46	8	213.166	1ch198357255	1.33	0.37	7.215	7.119	6.949	1; 3
qDTS-3	3.07	6.08	3.8	179.152	3ch195814817	−0.32	−1.03	6.257	6.186	6.322	2; 3; 4
qDTS-4	4.07	7.96	13	177.898	4ch179647258	0.7	−2.31	6.715	6.415	6.575	2; 3
qDTS-5	5.01	8.35	3.4	48.343	5ch5578080	0.46	1.15	7.128	7.197	7.036	1; 3
**qDTS-6**	**6.01**	**6.02**	**9.9**	**27.25**	**6ch85961881**	**1.41**	**−0.78**	**6.786**	**6.567**	**6.505**	**2; 3; 4**
qDTS-7	7.05	12.17	5.2	267.416	umc2333	0.63	0.31	7.144	7.112	7.018	1; 4
qDTS-8.1	8.01	8.75	3.5	87.423	8ch7301310	0.8	0.42	7.162	7.124	7.002	1; 4
qDTS-8.2	8.09	6.04	3.8	287.835	8ch173447681	−0.39	−1.3	6.250	6.159	6.328	1; 4

^a^ Integrated QTL name composed by the trait code followed by the LG number in which the QTL was mapped and a decimal if more than one integrated QTL is detected for the same trait in the same LG

^b^ Linkage group and bin position of the nearest flanking marker to the highest LOD peak

^c^ Maximum LOD peak in the integrated QTL

^d^ Maximum phenotypic variation explained in the integrated QTL

^e^ LOD peak position of the maximum LOD peak in the integrated QTL

^f^ Nearest flanking marker to the maximum LOD peak in the integrated QTL

^g^ Negative and positive values refer to CO354 and CO441 as the origin of the beneficial allele, respectively

^h^ Negative values indicate that the heterozygotes are more resistant than the respective mean between the two homozygotes

^i^ Mean of the distribution of quantitative trait associated with the CO354 genotype

^l^ Mean of the distribution of quantitative trait associated with the CO441 genotype

^m^ Mean of the distribution of quantitative trait associated with the heterozygote genotype. When no dominance was fitted mu_H correspond to the mean of the sum among the average of the two homozygotes

^n^ Year, sowing time and inoculation technique in which the QTL was detected: 1 = 2011A; 2 = 2011B; 3 = 2012A; 4 = 2012B; F = side-needle inoculation; T = toothpick inoculation

No integrated QTL was found in the LG 10. DTS QTLs were mapped on the LGs 1, 3, 5–8. FER QTLs were associated to the largest number of LGs, being located on all LGs excluding LG 10, whilst FB1 contamination QTLs mapped on LGs 1–9, with the only exception of LG 8. Eight integrated QTLs were in common between FER and FB1 contamination traits, positioned on the LGs 1, 2, 3, 6, 7 and 9 (Table 2; Additional file 6: Figure S2). Moreover, the qFER-6 and qFB1-6.1 integrated QTLs co-mapped with a QTL for DTS (qDTS-6) (Table 2; Additional file 6: Figure S2).

The average value of the phenotypic variation explained by each of the integrated QTLs, considering the maximum R^2^ value, was the highest for FER (R^2^ = 9.4%), followed by DTS (R^2^ = 6.7%) and FB1 contamination (R^2^ = 6.6%).

### QTLs for FER, FB1 contamination and DTS

Among the QTLs for FER, 11 were detected in both 2011 and 2012 and only four in either year: qFER-3, qFER-4, qFER-8 and qFER-9.1 (Table 2). The phenotypic variation explained by QTLs (R^2^) ranged between 4.5% (qFER-2.1) and 18.7% (qFER-9.2). The highest percentage of variation was explained by qFER-9.2, which was detected in 2011B_F and _T and 2012A_T. The resistant parent CO441 carried the beneficial alleles (decreasing FER) in the cases of FER QTLs on LG 2, qFER-6, qFER-8 and qFER-9.4 while the susceptible parent CO354 contributed favorable alleles for qFER-1, qFER-3, qFER-4, qFER-5, qFER-7 and qFER-9.1- qFER-9.3 (Table 2). The beneficial alleles were partially dominant over the susceptibility alleles in qFER-2.1 and qFER-2.4, qFER-5, qFER-6, qFER-7, qFER-9.1 and qFER-9.3 and were completely dominant in qFER-2.3.

Most of QTLs for FB1 contamination were detected in both sowings of 2012 (qFB1-1.3, qFB1-3, qFB1-4.1-qFB1-4.3, qFB1-5, qFB1-9.2 and qFB1-9.3) and only four QTLs (qFB1-1.2, qFB1-6.2, qFB1-7.2 and qFB1-9.4) were stable across years (Table 2). The minimum R^2^ value was associated to qFB1-6.2 (R^2^ = 2.9) and the maximum value to qFB1-2 (R^2^ = 17.2). Most of the QTLs for FB1 contamination derived from the susceptible parent (qFB1-1.1, qFB1-1.2, qFB1-3, qFB1-4.1, qFB1-4.2, qFB1-4.3, qFB1-5, qFB1-7.1, qFB1-7.2, qFB1-9.3 and qFB1-9.4), with a partial dominant effect of the beneficial alleles (reduction of FB1 contamination) in qFB1-4.2, qFB1-4.3, qFB1-5, qFB1-7.1, qFB1-7.2, qFB1-9.3 and qFB1-9.4. The resistant parent contribution (qFB1-1.3, qFB1-2, qFB1-6.1, qFB1-6.2, qFB1-9.1 and qFB1-9.2) revealed a partial dominant effect of the beneficial alleles in all cases (Table 2).

All nine integrated QTLs for DTS were stable across years, although only qDTS-3 and qDTS-6 were stable also across sowing times. The lowest R^2^ value was associated to qDTS-5 (3.4) and the highest to qDTS-4 (13). Only two DTS QTLs derived from the susceptible parent (qDTS-3 and qDTS-8.2), showing partial dominance of the favorable alleles. The resistant parent contributed to the QTL for earliness in DTS in the other seven cases, with effect of partial dominance in qDTS-1.1, qDTS-4 and qDTS-6 (Table 2).

### QTLs in common to FER and FB1

According to the 2-LOD confidence intervals, eight QTLs for FER and FB1 contamination mapped in similar positions of the maize genome and were located on LGs 1, 2, 3, 6, 7 and 9. Most of these overlapped regions explained the highest percentage of phenotypic variation. This is the case of qFER-1 and qFB1-1.1 (R^2^ = 11 and 6.1, respectively), qFER-6 and q-FB1-6.1 (R^2^ = 13.6 and 3.9, respectively), qFER-7 (R^2^ = 17.5) which co-mapped with both qFB1-7.1 (R^2^ = 12.8) and qFB1-7.2 (R^2^ = 12.4). The qFER-7, qFB1-7.1 and qFB1-7.2 QTLs showed great stability across years, sowing time and inoculation technique, since they were not detected only in 2011B_F and 2012B_F (qFB1-7.1 was detected also in 2011A_F, but with a LOD (3.6) slightly below the significance threshold). In this case the beneficial partially dominant alleles of qFER-7 and qFB1-7.1-7.2 QTLs were carried by the susceptible parent (Table 2).

The qFER-3 QTL (R^2^ = 7.5) was detected in both sowings of 2011 and co-mapped with the qFB1-3 QTL (R^2^ = 5.6), determined in both sowings of 2012 and in 2011A_F with a LOD (3.0) lower than the significant threshold set by the permutation test (4.2). Interestingly, different regions of LG 9 showed associations for both FER and FB1 contamination, although with smaller percentage of explained phenotypic variation (average R^2^ = 5.6): qFER-9.1 co-mapped with qFB1-9.2, qFER-9.3 with qFB1-9.3 and qFER-9.4 with qFB1-9.4.

The qFER-2.4 and qFB1-2 QTLs, associated to the SSR bnlg1909, explained the 11.6 and 17.2% of the FER and FB1 phenotypic variation, respectively. Although qFB1-2 was detected only in 2011, qFER-2.4 was stable across years. Interestingly, the additive and dominance effects associated to both QTLs revealed that the resistant parent contributed to resistance carrying a partially dominant allele (Table 2).

### Candidate genes for FER resistance

In order to identify candidate genes for *Fusarium* resistance traits, we considered the 1-LOD confidence intervals of the integrated QTLs in common between FER and FB1 contamination: qFER-2.4 and qFB1-2 (which explained the highest phenotypic variation for FB1 trait), and qFER-7 and qFB1-7.1 and −7.2 (the most stable QTLs). In particular, we focused on differentially expressed genes (DEGs) found in a previous transcriptomic comparison of resistant CO441 and susceptible CO354 genotypes at 72 h after *F. verticillioides* inoculation [34]. The full list of DEGs included in the 2-LOD interval of the above mentioned regions and in the other six QTLs in common to both traits are reported in Additional file 7: Table S5.

Ten DEGs were located within the qFER-2.4/qFB1-2 1-LOD confidence interval and 125 for qFER-7/qFB1-7.1 and -7.2 (Additional file 7: Table S5). Among these, DEGs were firstly selected on the basis of their expression levels and secondly on their known role in plant defense (Table 3). DEGs were subsequently divided in “constitutive”, if differentially expressed between uninoculated CO441 and CO354 control samples, and “modulated”, if differentially expressed between inoculated and control samples of either or both genotypes.

**Table 3:** Differentially expressed genes among CO441 and CO354 genotypes within QTL regions in common between *Fusarium* ear rot and fumonisin B1 contamination traits

Integrated QTL	LG: start-end position^a^	Gene_ID	Sequence description^b^	FC^c^	Transcr. regulation^d^
qFER-2.4 qFB1-2	2: 45192080-48148553	GRMZM2G031331	Barley *mlo* defense gene homolog3	R	Modulated
		GRMZM2G104843	Lipoxygenase 8	RTS	Modulated
		GRMZM2G331701	22.0 kDa class IV heat shock protein	RTS	Modulated
		GRMZM2G086971	Thioredoxin	RTS	Constitutive
		GRMZM2G053111	Serine threonine-protein kinase-like ccr4	ST	Modulated
qFER-7 qFB1-7.1 qFB1-7.2	7: 50591357-129971622	GRMZM2G461159	S-adenosylmethionine decarboxylase	MP	Modulated
		GRMZM2G477743	Monoglyceride lipase-like	MP	Modulated
		GRMZM2G093947	RNA-binding protein rbp37	MP	Constitutive
		GRMZM2G133613	*Avr9* elicitor response protein	R	Modulated
		GRMZM2G328877	Heat shock protein 70	RTS	Constitutive
		GRMZM2G139535	Heat shock factor-transcription factor 21	RTS	Modulated
		GRMZM5G813217	Heat shock protein 83-like	RTS	Modulated
		GRMZM2G153607	Early-responsive to dehydration stress-related protein	RTS	Modulated
		GRMZM2G139815	WRKY 74 transcription factor	ST	Modulated
		GRMZM2G334165	Cysteine-rich receptor-like protein kinase 10	ST	Modulated
		GRMZM2G025761	Transcriptional adaptor family protein	ST	Constitutive
		GRMZM2G123119	APETALA2/ethylene responsive element binding protein transcription factor	ST	Modulated
		GRMZM2G467943	APETALA2/ethylene responsive element binding protein transcription factor	ST	Constitutive
		GRMZM2G141219	APETALA2/ethylene-responsive transcription factor at1g16060-like	ST	Modulated
		GRMZM2G052667	APETALA2/ethylene responsive element binding protein transcription factor 102	ST	Modulated
		GRMZM2G092137	OCS element-binding factor 1	ST	Modulated
		GRMZM2G009045	Phosphate carrier mitochondrial-like	T	Modulated
		GRMZM2G075951	Carbohydrate transmembrane transporter	T	Constitutive
		AC234166.1_FG002	Hypothetical protein ZEAMMB73_317354	U	Constitutive

^a^ Linkage group and start and end positions in bp of the 1-LOD confidence interval

^b^ Putative gene annotation as automatically associated using Blast2GO software

^c^ Functional category: MP = metabolic process; R = resistance; RTS = response to stress; SM = secondary metabolism; ST = signal transduction; T = transport; U = unknown

^d^ Transcriptional level of the gene in uninoculated control and inoculated kernels at 72 h post inoculation of CO354 and CO441 genotypes found in [34]: constitutive = differentially expressed gene (DEG) among the CO441 and CO354 controls; modulated = DEG in either or both CO441 and CO354 genotypes after inoculation in comparison to controls

Among the ten DEGs in the 3.0 Mb 1-LOD interval associated to qFER-2.4 and qFB1-2, two genes showed different constitutive expressions between genotypes in the uninoculated controls and eight were specifically modulated upon infection (Additional file 7: Table S5). Beside genes with unknown function (GRMZM2G152141 and GRMZM2G179827), genes with low expression level (GRMZM2G072984 and GRMZM2G112039) and genes currently not known to be related to defense (GRMZM2G497438), five genes potentially related to *F. verticillioides* resistance were identified: a constitutively expressed thioredoxin (YPTM1) and the genes modulated upon inoculation, namely the lipoxygenase *LOX8*, a heat shock protein (*HSP*) (GRMZM2G331701) involved in response to stress, a *mlo* defense gene (GRMZM2G031331) and a serine threonine-protein kinase-like gene (GRMZM2G053111), related to resistance and signal transduction, respectively (Table 3). The genes involved in response to stress showed large differential expression among genotypes, with the thioredoxin gene constitutively expressed up to 89 times more in CO441 in comparison to CO354, and the *HSP* induced after inoculation more than twice in the susceptible genotype (Table 3; Additional file 7: Table S5). *F. verticillioides* inoculation led to *LOX8* induction up to 2 and 2.4 times in the susceptible and resistant genotype, respectively.

A total of 125 DEGs associated to qFER-7, qFB1-7.1 and −7.2 were present in the 79.4 Mb interval. One third of them (43 DEGs) were differentially regulated among CO441 and CO354 genotypes at the constitutive level (Additional file 7: Table S5) and a large part were hypothetical proteins (20) or had unknown function (23). From the total list of 125 DEGs, we focused on 19 based on expression values and known function (Table 3). Among the stress-related proteins, the early-responsive to dehydration stress-related protein (GRMZM2G153607), two *HSPs* and a transcription factor regulating their activation (GRMZM2G139535) were included in this interval. The *Avr9* elicitor response protein gene (GRMZM2G133613), involved in resistance, was modulated in both genotypes after inoculation, as well as a phosphate carrier mitochondrial-like (GRMZM2G009045), which increased 455 and 188 times after inoculation in the resistant and susceptible genotypes, respectively. Genes involved in signal transduction included a cysteine-rich receptor-like protein kinase 10-like (GRMZM2G334165) and a WRKY transcription factor (GRMZM2G139815), both modulated after inoculation, and the constitutively expressed transcriptional adaptor family protein (GRMZM2G025761). Four APETALA 2/ethylene responsive element binding protein transcription factors (AP2/ERF) were constitutively expressed (GRMZM2G467943), or regulated after pathogen inoculation in both genotypes (GRMZM2G123119 and GRMZM2G141219), or induced only in the susceptible genotype (GRMZM2G052667). The OCS element-binding factor 1 (GRMZM2G092137) was significantly induced (up to 5 times) after inoculation in CO441. Other genes differentially expressed encoded RNA-binding protein rbp37 (GRMZM2G093947) and a carbohydrate transmembrane transporter (GRMZM2G075951), which were 227- and 153-fold more constitutively expressed in the resistant line, respectively. The monoglyceride lipase-like (GRMZM2G477743) redoubled its expression value after inoculation in the resistant line, while the S-adenosylmethionine decarboxylase (GRMZM2G461159) increased four-fold its expression. Notably, the hypothetical protein ZEAMMB73_317354 (AC234166.1_FG002) was constitutively expressed 1,400-fold in the resistant genotype.

A larger number of candidate genes for resistance could be obtained considering the 2-LOD confidence intervals around the LOD peak (Additional file 7: Table S6). Whilst 2-LOD intervals of qFER-7 and qFB1-7.1 and −7.2 corresponded to 1-LOD extremes, the 2-LOD region associated to qFER-2.4 and qFB1-2 spanned 40 Mb and included the resistance-related hypersensitive-induced response protein (GRMZM2G157869) and the RPM1-interacting protein 4-like (GRMZM2G027272) and the stress-related ascorbate peroxidase (APX, GRMZM2G120517).

In the other QTLs regions in common between FER and FB1 traits some genes are noteworthy because of their expression values: the stress related-like protein interactor (GRMZM2G458718) in qFER-1/qFB1-1.1, the glutathione S-transferase (GRMZM2G019090) in qFER-3/qFB1-3, the PLATZ transcription factor family member (GRMZM2G006585), the ethylene-responsive factor-like protein 1 (GRMZM2G053503) and the *HSP* 90 (GRMZM2G063988) in qFER-9.1/qFB1-9.2, the *HSP70-2* (GRMZM2G324499) and the APX (GRMZM2G054187) in qFER-9.3/qFB1-9.3, the vicilin-like antimicrobial peptide 2–3 (GRMZM2G078441) in qFER-9.4/qFB1-9.4 (Additional file 7: Table S6).

## Discussion

### FER disease incidence and FB1 accumulation in the F_3_ population

FER and fumonisin contamination are influenced by many environmental factors and test sites with consistently high disease pressure are required for genetic analysis of these traits [37]. In general, low rainfall and high temperature around flowering, and high rainfall or high temperature just before harvest, were found to favor fumonisin contamination [2]. In the present study, temperatures and relative humidity were significantly different in the 2 years of phenotyping, but no significant changes occurred between the two sowing times within the same year. In particular, both flowering and post-inoculation periods in 2012 were characterized by significantly higher temperatures and lower relative humidity compared to 2011.

Significant differences among groups determined by years, inoculation technique and sowing times were recorded for both FER severity and FB1 contamination. Consistent with climatic patterns, FER severity and FB1 contamination were significantly higher in 2012B, when higher temperatures and lower relative humidity also accelerated flowering (DTS).

The distributions of FER and FB1 traits in the F_3_ population revealed the presence of transgressive segregants, i.e., families performing outside the parental range. Transgressive segregants were observed in populations screened for FER and fumonisin contamination [14], but also for *Gibberella* ear rot (GER), caused by *F. graminearum*, and deoxynivalenol (DON) and zearalenone (ZEA) contamination [38–40]. This phenomenon occurs because of the accumulation of favorable and unfavorable alleles originating from both parental lines (additive effect) and/or epistatic interactions. In the present study, identification of offspring outperforming the resistant parent CO441 provides a starting point for improvement of *Fusarium* and fumonisin resistance in breeding programs.

The low positive significant correlations (*r* = 0.16–0.30), detected in 2011 and 2012 between flowering time (DTS) and FB1 contamination, suggested that in few maize genotypes late flowering is associated with higher fumonisin content. On the other side, significant low positive correlations between DTS and FER were found only in 2012, suggesting that fumonisin content is more influenced than disease severity by the plant developmental stage. It was demonstrated that early flowering hybrids normally show reduced ear rot severity and mycotoxin contamination [36]. Nonetheless, higher temperatures, occurred in 2012, might affect both flowering and the FB1 content. Further analyses are needed to establish if the correlation between ear rot and silking date arises from linkage between resistance genes and early flowering genes.

Since under natural conditions the pathogen is not evenly distributed in the field, artificial inoculation is key to ensure its equal distribution [41]. In our study, plants were artificially inoculated with either the side-needle or the toothpick inoculation technique. Both techniques allowed to evaluate kernel resistance, although the parents were initially screened for silk channel resistance [42] and the correlation between the two traits was low in certain genotypes [36]. Moreover, injection through the ear husks resulted in higher levels of fumonisin concentration and infection severity [7, 43, 44]. In this study, both inoculation techniques allowed a reliable differentiation of genotypes for kernel resistance according to disease level and fumonisin content. Regarding the comparison between the two inoculation techniques, they significantly affected FER severity more than sowing date and year: disease symptoms were lower in plants infected with the toothpick, but similar disease levels were found between 2011A, 2011B and 2012A. Lower FER severity in toothpick-inoculated ears may be due to slower progress of pathogen growth when inoculated as mycelium in comparison to conidia, or to smaller diameter of the inoculation punch. On the other hand, fumonisin content was not clearly affected by the use of either inoculation technique. Anyway, ease of use and similar efficacy in genotype screening make the side-needle inoculation technique preferable to the toothpick.

In contrast to most plant diseases, which can be simply visually rated, the evaluation of fumonisin contamination resistance requires time-consuming and expensive toxin assays. The high phenotypic correlation (ranging between 0.72 and 0.81) between FER severity and FB1 contamination, determined in this study, demonstrated that analyses for mycotoxin content are only rarely needed. Robertson and coworkers [14] determined lower phenotypic correlations between these traits in two populations (0.40 and 0.64), but the strong genotypic correlations (0.96 and 0.87) suggested that selection for reduced ear rot should frequently identify lines with reduced fumonisin contamination. Therefore, in breeding programs, selecting against ear rot may be a useful strategy for selecting genotypes with lower susceptibility to fumonisin contamination.

### QTLs for FER and FB1 contamination resistance

Fifteen QTLs for FER and seventeen for FB1 content were detected in the CO441xCO354 F_3_ population (integrated QTLs). All identified QTLs had relatively small effects, explaining on the average 7.9% of the phenotypic variation, with values ranging between 2.9 and 18.7%, in agreement with previous results [15, 16]. The existence of favorable alleles for FER and FB1 contamination resistance in both parents can at least in part explain the presence of transgressive segregants in the progeny, with higher resistance or susceptibility than the CO441 and CO354 parents, respectively. Additional mechanisms, such as epistasis and overdominance, may also play a role in this respect. While both parents carried equally the beneficial alleles for FER resistance, CO354 contributed to most of the FB1 resistance QTLs and was associated to some of QTLs explaining the largest proportion of the phenotypic variation, as qFER-7.1 (R^2^ = 12.8) and qFB1-7.2 (R^2^ = 12.4). Dominance effects were detected for most of the alleles contributing to resistance, except qFER-1, qFER-2.2, qFER-3, qFER-4, qFER-8, qFER-9.2, qFER-9.4, qFB1-1.1, qFB1-1.2, qFB1-3 and qFB1-4.1.

Comparing the positions of QTLs for FER and FB1, few correspondences were found with previously identified QTLs [15–18], probably due to the different sources of resistance used in previous studies [15–18]. Indeed, limited consistency across populations was already noted by other authors evaluating different populations sharing a common parent [15], or completely different parents [16]. In particular, only three FER and two fumonisin contamination resistance QTLs mapped to similar positions in the GEFR (GE440xFR1064) and NCB (NC300xB104) populations [16].

Surprisingly, a high correspondence was detected with QTLs related to *F. graminearum* resistance, causing GER and the contamination of maize grains with ZEA and DON mycotoxins [38–40]. The parental lines employed in the present study were initially selected for *F. graminearum* resistance, but showed analogous levels of resistance to FER and common smut (*Ustilago zeae*) [42]. Indeed, a cross with parent CO441 was recently used to detect QTLs related to GER resistance [45]. Resistant sources to multiple maize pathogens, such as *F. verticillioides*, *F. graminearum* and *A. flavus* were found, suggesting the presence of a common genetic mechanism regulating resistance to different diseases [6, 10, 13]. In selected resistant and susceptible genotypes for fumonisin contamination, the genotypic correlations between FER, *Aspergillus* ear rot (AER), fumonisin and aflatoxin contamination were always greater than the phenotypic correlations, highlighting again the central role of the environmental factors on the phenotype [6].

Interestingly, eight QTL positions detected in this study overlapped between FER and FB1 contamination, suggesting the existence of genetic mechanisms controlling susceptibility/resistance to both traits. The high phenotypic correlations between the two traits found in this study (0.72–0.81) and the high genetic correlations found in the GEFR and NCB populations (0.96 and 0.87, respectively) [14], demonstrate feasibility of MAS of genotypes showing resistance to both FER and FB1 contamination.

Among the overlapping QTLs between the two traits, the CO354 line contributed to FER resistance and low FB1 contamination in QTLs located on the LGs 1, 3, 7 and 9 (qFER-9.3 and qFB1-9.4), while CO441 line carried the favorable alleles on LG 2 and LG 6. The overlapping qFER-7 and qFB1-7.1 and -7.2 QTLs were stable across years and sowings, with R^2^ ranging from 12.4 to 17.5%. The qFER-3 and qFB1-3 QTLs were detected in different years, explaining 7.5 and 5.6% of the phenotypic variation, respectively. To our knowledge, these QTLs on LG 3 and LG 7 were never mapped previously neither for resistance to *F. verticillioides*, nor *F. graminearum* or *A. flavus*. These genomic regions, which derived both from CO354 line and are associated to resistance to both traits across years, are promising targets for the improvement of *F. verticillioides* resistance in maize.

LG 2 showed numerous consistent QTLs localized in four regions: qFER-2.1, qFER-2.2, qFER-2.3 and the overlapping qFER-2.4 and qFB1-2, both associated with SSR bnlg1909. The latter QTLs, detected in both years, explained a large amount of the phenotypic variation, with R^2^ for FER and FB1 contamination of maximum 11.6 and 17.2%, respectively. Similar bin positions were related to QTLs associated to GER resistance and ZEA and DON contamination reduction [38–40]. In particular, bnlg1909 was positioned, according to the IBM2 2008 Neighbors2 map, less than 4 cM from umc1259, the nearest flanking marker of QTLs for GER, ZEA, DON resistance in a UH007xUH006 doubled haploid line population [39] and co-located with bnlg108, flanking marker in GER resistance QTLs in UH009xUH007 doubled haploid lines [40] and in CO387xCG62 RILs [38]. Considering the stability of this QTL, which determined both FER and FB1 contamination, but also resistance towards *F. graminearum*, the genomic region around SSR bnlg1909 is an interesting target for further dissection and candidate gene identification.

LG5 showed the presence of QTLs for DTS (qDTS-5), FER (qFER-5) and FB1 contamination (qFB1-5), explaining respectively 3.4, 10.0, and 7.7% of the phenotypic variation. Moreover, qFER-5 was located near a QTL for DON contamination reduction in bin 5.02 between bnlg565 and umc2167 markers [39], as well as QTLs for aflatoxin contamination in bin 5.01, between bnlg143 and bnlg565 markers [46], and in bin 5.03, associated to bnlg1046 [47].

The qFB1-4.2 QTL was localized in bin 4.05, and 4.04–4.05 bins represent a region of clustered QTLs for FER resistance [18], GER resistance [39] and aflatoxin resistance in several populations [48]. Furthermore, SNPs associated with resistance to GER in a CO441xB73 RIL population were located close to qFER-1/qFB1-1.1, qFER-2.2, qFB1-9.1 and qFER-9.3/qFB1-9.3 [45]. Similarly, qFB1-1.3, qFB1-4.1, qFER-9.1 and qFB1-9.2 localized in bin positions corresponding to QTLs for GER resistance, ZEA and DON contamination reduction [39, 40].

A QTL related to flowering (qDTS-6) was co-located with a QTL for fumonisin contamination (qFB1-6.1) and a QTL for FER (qFER-6), detected in late sowings of both years with an R^2^ of 13.6%. Also qDTS-6 showed great stability across years and sowings, explaining 9.9% of the phenotypic variation, in contrast to qFB1-6.1 that was uncovered only in 2012A with a R^2^ equal to 3.9%. Association of disease resistance traits with maturity-related QTLs was reported before [49], reflecting the low positive correlation determined between the two traits. Since the resistant parent contributed to these QTLs, a pleiotropic gene effect between earliness in flowering, FER and FB1 contamination reduction may exist.

Some QTLs detected in our study overlapped with genomic regions previously associated with responses to other diseases. This is consistent with previous observations that QTLs for different disease resistances have a tendency to map to similar genome locations, implying the existence of common genetic mechanisms regulating these traits [49]. A meta-QTL analysis, comprehending 87 individual QTLs for FER, GER and AER resistance derived from 14 different studies, revealed the presence of 29 clusters of QTLs (meta-QTLs), mainly localized on chromosome 3, 4 and 5 [50]. In our study, QTLs associated to *F. verticillioides* response on LG 4 and LG 5 overlapped or were adjacent to QTLs related to *F. graminearum* and *A. flavus* resistance, confirming the existence of clusters of resistance QTLs. Fine scale-genetic mapping will be necessary to distinguish linked QTLs, such as those in a “resistance cluster”, from pleiotropic QTLs that influence resistance to multiple fungi. Interestingly, the Canadian CO387 inbred, developed for *F. graminearum* resistance as our parents, was the source that mostly contributed to resistance against FER, GER and AER in the meta-QTL analysis [50].

Recently, two genome-wide association studies were conducted on the maize core diversity panel inbred lines to detect allele variants associated with increased resistance to FER [29, 30]. Only two disease-associated SNPs mapped in similar positions to QTLs detected in our study: the first SNP, physically positioned at 151,295,233, was located within the 1-LOD interval of the overlapping qFER-9.4/qFB1-9.4, and the second SNP was less than 5 Mb from the SNP 2ch2,672,212 associated to qFB1-4.1.

The multifactorial nature of *Fusarium* and fumonisin contamination resistance poses challenges to their genetic improvement through MAS, which is most effective when few moderate-large effect QTLs and consistent effects across breeding populations can be identified [51]. However, MAS appears the most suitable technique for improving agronomically performing lines because of the high costs related to phenotyping. Further studies using different populations derived from the CO441xCO354 cross and increasing the tested environments, will confirm the presence, the location and effects of FER and FB1 contamination QTLs, minimizing the environmental effects on these traits.

In conclusion, the QTLs mapped in this study are an important source for further studies on FER and FB1 contamination. The choice of the parents, with contrasting levels of FER and FB1 contamination, allowed an adequate segregation of these traits in the study population. Moreover, the choice of a high-yielding resistant parent (CO441) provides an ideal basis for the selection of resistant hybrids with good agronomic performance in future breeding programs.

### Candidate genes for FER resistance

Candidate genes for maize resistance to FER have been proposed in transcriptomic studies comparing the response of susceptible and resistant maize lines to *F. verticillioides* infection, suggesting that resistance of some genotypes may be mainly due to the constitutive expression of defense mechanisms [31–34, 52]. In this study, the genomic regions spanning QTLs of particular interest were scanned for the presence of DEGs resulting from an RNA-Seq experiment led on the same two parents at 72 h post *F. verticillioides* inoculation [34]. Two genomic regions in common between FER and FB1 traits were firstly considered as potential sources of genes for *F. verticillioides* resistance: qFER-2.4 and qFB1-2, which explained the highest phenotypic variation for FB1 trait, and qFER-7 and qFB1-7.1 and −7.2 which showed the greatest stability across years and sowings. The 1-LOD confidence intervals of these regions harbored in total 135 DEGs, which could be considered potential candidates for *F. verticillioides* resistance and about one third of them (48/135) has unknown function. Among DEGs associated to these intervals of interest, 24 were analyzed in more detail based on high expression levels and known defensive role (Table 3).

In the region containing the qFER-2.4 and qFB1-2 QTLs, three genes related to stress response could be considered as valuable candidates for resistance: *YPTM1*, *LOX8* and 22 kDa *HSP. YPTM1* was constitutively present in uninoculated controls of the resistant line and thioredoxins are known to protect plant tissues from oxidative damage produced by reactive oxygen species, triggered after pathogen recognition. In maize, *LOX8*, which resulted up-regulated in both genotypes after *F. verticillioides* inoculation [34], is the only identified family member responsible for the production of jasmonic acid (JA) [53], an oxylipin that mediate the response to necrotrophic pathogens, together with ethylene (ET) [54]. Oxylipins are compounds involved in signaling and are implicated in plant-pathogen interactions and regulate mycotoxin production [55]. The role of *LOXs* in maize-*F. verticillioides* interaction and other pathogen cross-talks was demonstrated by the use of maize mutants [56–59]. Moreover, *LOXs* and other genes involved in oxylipin biosynthesis were induced earlier and more strongly in the resistant CO433 inbred in comparison to CO354 after *F. verticillioides* inoculation [60]. In the susceptible line, a gene coding for 22 kDa *HSP*, involved in protein folding and stabilization, was specifically induced after inoculation [34]. Similar results were observed in several susceptible and resistant maize inbreds after *F. verticillioides* and *A. flavus* inoculation [61, 62]. The candidate genes in the region harboring the qFER-2.4 and qFB1-2 QTLs should be considered for further breeding studies which aim at developing highly resistant hybrids to multiple fungal pathogens, due to the proximity with QTLs for *F. graminearum* resistance [38–40].

In the larger genomic region containing qFER-7 and qFB1-7.1 and −7.2 QTLs, several HSPs, constitutively expressed level or modulated after inoculation, could be considered as candidates for resistance. Among transcription factors, WRKY74 was *F. verticillioides* responsive in both genotypes. Numerous members of the WRKY family are associated to the plant immune response, starting the transcription of pathogenesis related genes [63, 64]. Several members of AP2/ERF transcription factor superfamily mapped in this region and they are known to be induced by biotic and abiotic stresses and bind to the promoter regions of stress responsive genes, including defense-related genes, pathogenesis-related (PR) genes, osmotin, chitinase and β-1,3-glucanase [65]. The S-adenosylmethionine decarboxylase, a key enzyme for the synthesis of the polyamines, is involved in pathogen response [66] and it was strongly up-regulated in the CO441 genotype [34]. A higher constitutive expression in the resistant genotype of a carbohydrate transmembrane transporter, in comparison to CO354, might confirm the role of sucrose in mounting the defense response in the resistant plant by avoiding its reallocation forced by pathogens [67, 68].

In general, the parental contribution to a QTL reflected the expression levels of the harbored candidate genes, although exceptions were found. The expression values of the candidate genes in parents both before and after inoculation are provided as fragments per kilobase of transcript per million fragments mapped (FPKM) in E-H columns of Additional file 7: Table S5. For example, the resistant parent contributed to qFER-2.4 and qFB1-2 QTLs and among the 10 DEGs within the QTL interval, three candidate genes (including *YPTM1*) showed significantly higher constitutive expression in the resistant line in comparison to the susceptible one. *LOX8* was induced in both genotypes after inoculation with a greater fold change in the resistant line and two other genes were modulated after inoculation only in the resistant line (Additional file 7: Table S5). Moreover, the defense mechanism may be identified in some cases as the down-regulation upon inoculation of the genes involved in susceptibility rather than the up-regulation of resistance genes. An example is given in qFER-2.4 and qFB1-2 QTLs by a 22.0 kDa *HSP* (GRMZM2G331701), which did not change after inoculation in the resistant line, contributing with beneficial allele to resistance, but it was significantly up-regulated in the susceptible line (Additional file 7: Table S5). Smaller map intervals will allow a more precise identification of the genes involved in resistance and resolve the cases in which the parental contribution with beneficial alleles do not correspond to the observed gene expression levels.

Finally, a genome-wide association study identified a FER-associated SNP within the intron region of GRMZM2G178880, positioned at 0.7 Mb from the cofactor marker associated to qFER-9.4/qFB1-9.4 [29]. This gene, coding for a cellulose synthase-like family A protein, was not differentially expressed at 72 h post inoculation between the CO354 and CO441 parents [34] and, due to the intronic position of the polymorphism, it is more likely that it is in linkage with the causal variant and not the causal variant itself [29]. The 1-LOD interval of qFER-9.4/qFB1-9.4 harbors 33 DEGs, which could be considered candidate genes for resistance (Additional file 7: Table S5). In particular, the vicilin-like antimicrobial peptides 2–3 (AMP2-3, GRMZM2G078441) was induced after inoculation in the CO441 line reaching extremely high expression levels (FPKM = 616.42). Moreover, the FER-associated SNP was distant less than 4 Mb from the 1-LOD interval of the qFER-9.3/qFB1-9.3 QTLs, which included numerous stress-related proteins, as APX (GRMZM2G054187 and GRMZM2G054300), the *IN2-1* protein (GRMZM2G162486) and *HSP*s (GRMZM2G366532, GRMZM2G324499 and GRMZM2G024718) (Additional file 7: Table S5). Therefore these adjacent genomic regions on LG 9 could represent interesting sources of allelic variation for FER and FB1 contamination resistance in maize.

## Conclusions

SSR markers and GBS were applied in this study in order to identify QTLs associated to FER and FB1 contamination in a biparental mapping population. As well as mapping small effect QTLs for individual traits, we were able to uncover QTLs in common between FER and FB1 resistance traits making possible the selection of maize genotypes showing both low disease severity and low fumonisin contamination. Noteworthy are the overlapping qFER-2.4 and qFB1-2 QTLs, carried by the CO441 parent, which could find a direct application in maize breeding programs focused on improvement of *F. verticillioides* resistance, since this line is an agronomically performing inbred. The QTLs detected in this study were in some cases located close to QTLs for resistance to other mycotoxigenic fungi, suggesting their use for selection of lines resistant to multiple ear rots. Finally, candidate genes for resistance to *F. verticillioides* were identified combining previous transcriptomic data with QTL mapping, providing a set of genes that could be further studied to evaluate their usefulness in MAS.

## Methods

### Plant material

Two maize genotypes with contrasting phenotypes for resistance to FER were used: the resistant line CO441 and the susceptible line CO354 [13, 31, 32]. Both lines were obtained by the Eastern Cereal and Oilseed Research Centre, Agriculture and Agri-Food Canada (Ottawa, Canada) and were maintained by sibling at the Department of Sustainable Crop Production in Piacenza (Italy).

A segregating population was generated from the cross CO441xCO354. Resistant inbred CO441 (♀) was crossed once to the susceptible inbred CO354 (♂) to produce F_1_ seed. A population of 188 F_3_ families was developed by self-pollinating the F_1_ and F_2_ progenies. In 2011 and 2012, F_3_ families and parents were grown in Settala, Milan, Italy, in a randomized complete block design. Two sowing dates were used in each year: April 20^th^ and May 11^th^ 2011, April 28^th^ and May 11^th^ 2012. The early sowing trial is referred to as “A” and the late one as “B”. The experimental unit was the family.

Plots consisted of 25 plants planted into 3 m rows spaced 80 cm apart. Plots were hand-thinned to leave one plant every 20 cm and standard cultural practices were followed. Ten plants for each plot were hand self-pollinated and inoculated with *F. verticillioides* and the remaining plants were allowed to open-pollinate and used as controls for the natural infection evaluation.

Mid-silk dates (day from the sowing date when 50% plants in a plot showed emerged silks) were recorded and referred as “day to silking” (DTS). Flowering occurred in the period from June 24^th^ to July 07^th^ for 2011A, from July 09^th^ to 25^th^ for 2011B, from June 28^th^ to July 12^th^ for 2012A and from July 04^th^ to 20^th^ for 2012B.

Weather conditions, including rain (mm), maximum and minimum temperatures (°C) and relative humidity (%), were recorded daily in the period occurring from flowering to harvest of both 2011 and 2012 by a weather station placed in Rodano (MI), 5 km from the testing fields.

### Inoculum and mycelium production

*F. verticillioides* ITEM 294, a prolific producer of fumonisins, was used for kernel inoculations. The strain was obtained from the Institute of Sciences and Food Production, National Research Council, Bari, Italy. Cultures were maintained on Petri dishes (90 mm diameter) in Potato Dextrose Agar (PDA) and incubated at 25 °C with a 12 h photoperiod in the dark for 14 days.

For the side-needle inoculation method (F), conidia were collected by rinsing cultures with sterile water, scraping the agar surface with a scalpel and filtering the conidia suspension through sterile cloth. Spore suspension was adjusted to a final concentration of 3.5 × 10^6^ conidia/ml based on microscopic counts using a Bürker chamber.

For the toothpick inoculation method (T), doubled sterilized toothpicks were radially disposed on Petri dishes containing PDA and a tassel of the fungal inoculum was placed in the centre of the plate. Cultures were incubated at 25 °C for 14 days in the dark, in order to let the mycelium grow and cover the toothpicks.

### Inoculation and disease severity screening

Kernel resistance to FER was evaluated for the parents and for 188 F_3_ families in field trials. Inoculations with *F. verticillioides* were performed 15 days after pollination (DAP). For each plot, 5 ears were inoculated using the toothpick method (T), 5 ears using side-needle method (F) and 5 ears were non-inoculated and taken as controls for evaluating natural infection levels. For statistical analysis, ears inoculated with the same method were considered as replicates.

The side-needle inoculation device consists of three 250 mm-long needles mounted on a plastic handle. Pins were dipped in conidial suspensions of 3.5 × 10^6^ microconidia/ml and the tool was pressed through the husks sideways and into the center of the ear, penetrating the kernels to a depth of 5–10 mm. In the same way, toothpicks covered by mycelium were inserted for few seconds in the middle of the ear through the husks.

Disease severity was evaluated at maturity on hand-harvested and air-dried ears. Ear harvest was performed for both early and late sowing on September 15^th^ 2011 and September 11^th^ 2012.

Ear rot severity was evaluated visually based on a rating scale from 1 to 7, where 1 = no infection symptoms, 2 = 1–3%, 3 = 4–10%, 4 = 11–25%, 5 = 26–50%, 6 = 51–75% and 7 = 76–100% of the ear infected, respectively [69] (Additional file 8: Figure S3). The disease score of a particular plot, infected with F and T, was determined by averaging the scores of the five inoculated ears with either method. In the rating, ear rot severity under natural field condition was taken into account and subtracted from ear rot severity under inoculation.

FB1 contents were determined for each plot for both inoculation methods by Near-Infrared Spectroscopy (NIRS) [70]. Random 150–200 kernels belonging to five replicates were ground with a laboratory mill (Cyclotec^TM^ 1093 Sample Mill, FOSS) using a 1 mm mesh. Special attention was taken in the milling procedure in avoiding cross-contamination of different disease level kernels. Two subsamples of ground grains of nearly 4 g were measured for each plot using a model 6500 spectrometer (FOSS NIRSystems, Inc., Silver Spring, MD). FB1 content for a particular plot was determined by averaging the results from two subsamples that were treated as (technical) replicates. NIRS measurements were performed at CREA-MAC, Unità di ricerca per la maiscoltura (Bergamo, Italy) and processed according to the calibration equation developed in [70]. Values of FB1 given in the text are expressed in ppm (mg/kg).

### Phenotypic data analyses

Statistical analyses were performed using IBM SPSS statistic version 20. Tests of normality of the frequency distributions for FER and FB1 resistance and DTS traits were calculated by Shapiro-Wilk test. Significance of the differences between mean-rank values of FER, FB1 and DTS in the different sowing dates, inoculation techniques and years, were calculated through the nonparametric Friedman test (*P <* 0.05), followed by the post hoc Wilkoxon signed-rank tests (*P <* 0.05) with Bonferroni correction for multiple comparisons. Significance of correlations between traits was evaluated by calculating Pearson’s correlation coefficients.

Flowering period considered the days comprised between the first and last mid-silking day within each sowing time. The following period considered the days post-inoculation until harvest within each sowing time. Daily values for maximum and minimum temperatures, maximum and minimum relative humidity and rainfall corresponding to 2011 and 2012 and secondly relative to flowering and post-inoculation period in 2011A, 2011B, 2012A and 2012B were compared through rank-based nonparametric Kruskal-Wallis H test (*P <* 0.05) with Bonferroni correction for multiple comparisons.

### Molecular markers and population genotyping

In order to build a linkage map of the CO441xCO354 cross, we first built a framework map on the F_2_ progeny using published SSR markers. To further enrich the map in molecular markers, we decided to use the GBS approach [22]. Lyophilization and long storage of plant materials yielded DNA that was not appropriate for GBS, for which DNA integrity is essential. Because of the impossibility of using F_2_ leaves, we reconstructed the genotype of F_2_ individuals by pooling of 15–20 plants for each F_3_ progeny. In order to make sure that they adequately represented the genotype of the corresponding F_2_ plants, the F_3_ DNA pools were initially genotyped with 3 SSR markers (dupssr13, phi116, umc2118) and results were compared to SSR genotyping data previously obtained on the original F_2_ individuals (see below). In case of discrepancy between the two generations, a new F_3_ pool was constructed starting from independent F_3_ individuals or the relevant F_2_/F_3_ was excluded from map construction. A similar approach was used for the bulked segregants analysis [71] and was already successfully applied [72]. Therefore, the two maps were constructed on two consecutive generations. Using stringent criteria to retain only markers with consistent segregation, an integrated map was finally obtained based on 149 genotypes (see below).

### SSR genotyping

A total of 369 SSR primer pairs were selected according to their chromosomal positions on the reference map provided by Maize Genetics and Genomics Database (http://www.maizegdb.org) (Additional file 4: Table S3). Initially, these markers were screened on the two parents in order to identify polymorphisms. Polymerase Chain Reaction (PCR) products were separated by electrophoresis in 4% (w/v) agarose gels with the addition of 10,000X Sybr® Safe DNA Gel Stain (Invitrogen^TM^) and visualized at ultraviolet light.

DNA for SSR genotyping was obtained from young leaf samples of 157 F_2_ individuals and parent plants grown in the field at Settala, Milan, Italy. Leaves were harvested, freeze-dried, lyophilized and ground to fine powder. DNA extraction was performed from the powdered leaf material with DNeasy Plant Mini Kit system following the manufacturer’s instructions (Qiagen, Inc., Valencia, CA). Genotyping of F_2_ progenies and parents was performed at Parco Tecnologico Padano, Lodi, Italy, through indirect labeling [73]. Labeling was repeated for each SSR marker with two different fluorescent dyes (FAM and VIC, Applied Biosystems). The 10 μl reaction mixture contained 2 ng of genomic DNA, 40 nM of each PCR primer, PCR Buffer 1X (200 mM Tris-Cl, 500 mM KCl), 50 mM MgCl_2_, 10 mM dNTPs, 100 nM of labeled primer, TaqDNA polymerase 5U/μl (Promega, Madison, USA) and water to volume. Conditions of the PCR amplification are as follows: 94 °C × 5 min, 30 cycles × (94 °C × 30 s/56 °C × 45 s/72 °C × 45 s), 8 cycles × (94 °C × 30 s/53 °C × 45 s/72 °C × 45 s), 72 °C × 8 min. PCR products ran on the ABI 3730 Prism Genetic Analyzer (Applied Biosystems/Applera, Darmstadt, Germany) and data were analyzed by SoftGenetics’s Gene Marker (1.97 version). Filtering for markers with less than 30% missing data resulted in a SSR dataset including 95 markers.

### Genotyping-by-sequencing

Genomic DNA was extracted from fresh leaves using a NucleoSpin Plant II kit (Macherey-Nagel, Germany) following the manufacturer’s instructions. The DNA quantification was performed using PicoGreen (Invitrogen, Carlsbad, CA) and normalized to 10 μL of 10 ng/μL (100 ng total) in 96 well plates. The protocol [22] was followed to construct two libraries, containing 94 and 63 progenies, respectively plus the two parental lines (i.e., a total of 96 and 65 samples per library), for sequencing on Illumina HiSeq2000 platform (Illumina Inc., San Diego, CA). Each pool was run on a single flow cell lane using a 100 bp paired-end module on Illumina HiSeq2000 instrument at Parco Tecnologico Padano, Lodi, Italy.

Raw 100 bp reads from the two Illumina HiSeq lanes were processed with FastQC to check the overall quality of the sequence data. The reads were processed with a custom demultiplexer to remove the barcode adapters and assign each read to the corresponding sample. Next, the raw sequencing data for each sample were processed with Trimmomatic [74] to remove low quality bases and sequencing adapters, using the parameters ILLUMINACLIP:TruSeq3-PE.fa:2:30:10 LEADING:3 TRAILING:3 SLIDINGWINDOW:4:20 MINLEN:36. The filtered reads were mapped with BWA MEM [75] on the *Zea mays* genome (AGP v3.20) downloaded from Ensembl database and the resulting BAM files were sorted and indexed using SamTools v.0.1.19 [76]. The sorted BAM files were processed with Freebayes v9.9.2 [77], using parameters -m 30 -q 20 -R 0 -S 0, to perform the variation calling across all the samples. Filtering was applied to exclude INDELs and retain only SNPs that show polymorphism between the parental lines and missing data below 30%. SNP name were abbreviated with the chromosome number followed by the physical position on the reference genome (AGP v3.20).

### Genetic linkage map construction and QTL analysis

Genetic linkage map construction was based on a dataset of 149 genotypes (95 SSR markers and 1,700 SNPs), excluding genotypes missing >30% marker data. For each marker, the alleles of the CO354 and CO441 parents were encoded as A and B, respectively, in the data matrix used for linkage map construction and QTL analysis. Map construction was performed using the regression mapping algorithm of JoinMap 4.1 [78], using linkages with a recombination frequency smaller than 0.5 and a LOD larger than 0 and keeping other default settings. The recombination frequencies were transformed into genetic distances in centiMorgans (cM) through the Kosambi’s mapping function. The map was constructed including also markers exhibiting segregation distortion, but excluding markers mapping in incoherent positions in comparison with the reference genome. Finally, 342 SNPs and 41 SSRs were clustered into ten LGs.

QTL analysis was performed using the MAPQTL 6.0 software [79] for each phenotypic dataset (i.e., trait in 1 year, sowing time and inoculation technique). Following a permutation test for each trait (number of permutation fixed as 1,000), genome-wide LOD scores corresponding to *P* = 0.05 were considered as significance thresholds for the detected QTLs. According to this criterion, the estimated LOD threshold value of FER trait was 4.3 for 2012B_F, 4.1 for 2011A-B_F and 2012A_ F and 4.2 in the other cases. The genome-wide significance threshold for FB1 contamination trait was set to 4.1 for 2011A_T, 2011B_F-T, 2012A_F, to 4.2 for 2011A_F, 2012B_ T-F and to 3.9 for 2012A_T. The estimated LOD significance threshold for DTS was 4.2 in the year 2012, 4.1 in 2011A and 4.3 in 2011B. In a first analysis the Interval Mapping approach was used to estimate the QTL genomic interval and its contribution to the phenotypic variance. In order to detect which markers are significantly associated with QTLs and candidate as co-factors, the Automatic Cofactor Selection (ACS) was used. Multiple-QTL Mapping (MQM) was carried out in order to resolve the occurrence of multiple QTLs in the same LG. When a QTL associated with an ACS-validated cofactor marker showed a LOD lower than the significance threshold determined by the permutation test, this QTL was anyhow considered “significant” if (a) another significant QTL was determined in the same position for the corresponding phenotypic trait in another year, sowing time or inoculation technique and (b) the difference with LOD threshold was <0.5. Additive and dominance effects were calculated at the cofactor position by MapQTL, according to the formula (mu_A-mu_B)/2 and mu_H-[(mu_A + mu_B)/2], respectively, where: mu_A, mu_B and mu_H are the estimated mean of the distribution of the quantitative trait associated with the “A” genotype (CO354), “B” genotype (CO441) and “h” genotype, respectively. The maps of QTL positions, showing 1- and 2-LOD confidence intervals, were drawn using MapChart 2.1 software [80].

The overlapping of the 2-LOD confidence intervals within the same trait QTLs defined the new integrated limits of the QTL, thereafter referred to as the “integrated QTL”. QTL nomenclature for the integrated QTLs modified rules proposed in [81] and each QTL was designated with the code “qTc-LG.1”, where: q = quantitative trait; Tc = trait code (FER/FB1/DTS); LG = linkage group number; 1 = first chronological QTL for this trait reported on this LG, when more QTLs were detected in the same LG for the same trait.

### Source of candidate genes

The 1-LOD and 2-LOD confidence limits of the integrated QTLs overlapping for FER and FB1 contamination traits were considered for the search of candidate genes. The physical coordinates of the integrated interval limits were determined on the *Zea mays* genome (AGP v3.20; http://www.maizesequence.org/). Genomic positions of the integrated QTLs were compared with a list of DEGs between CO441 and CO354, derived from a previous RNA-Seq analysis of the maize ears at 72 h post *F. verticillioides* inoculation [34]. Gene annotation and functional categories were exported from Blast2GO [34].

## Additional files

Additional file 1:
**Figure S1.** Weekly means of A) temperature, B) relative humidity and C) weekly sums of rain precipitation in years 2011 and 2012. First week corresponds to the first 7 days of flowering in the early sowing of both 2011 and 2012. Last week corresponds to the week of the harvest day for both 2011 and 2012, in the early and late sowings. (PPTX 72 kb)
Additional file 2:
**Table S1.** Parent (CO441 and CO354) and F_3_ population mean values (± standard deviation, SD), F_3_ population range, median and mean-ranks for *Fusarium* ear rot severity (FER), fumonisin B1 (FB1) contamination and days to silking (DTS). Data are referred to early sowing (A) and late sowing (B) in 2011 and 2012, with side-needle (F) and toothpick (T) inoculation methods. (XLSX 13 kb)
Additional file 3:
**Table S2.** Pearson’s correlation coefficients between days to silking (DTS), *Fusarium* ear rot severity (FER) and fumonisin B1 concentration (FB1) of F_3_ families, measured with two inoculation methods (F = side-needle and T = toothpick), in early (A) and late (B) sowings of 2011 and 2012. (XLSX 10 kb)
Additional file 4:
**Table S3.** List of the 369 markers screened for polymorphism in the CO441 and CO354 parents and their use in map construction and QTL analysis. (XLSX 22 kb)
Additional file 5:
**Table S4.** Distribution of mapped SSR and SNP markers per linkage group (LG) on the reference genome Zea_mays.AGPv3.20.dna.genome.fa.gz (ftp://ftp.ensemblgenomes.org/pub/release-21/plants/fasta/zea_mays/dna/), total number of markers, average density and total genetic distance per LG. (XLSX 11 kb)
Additional file 6:
**Figure S2.** QTLs for *Fusarium* ear rot (FER), fumonisin B1 contamination (FB1) and days to silking (DTS). Marker names are listed on the right side of each LG and the corresponding genetic distances (in centiMorgan) on the left side. QTLs are drawn at the right of each LG and are represented as central bars indicating the 1-LOD confidence interval and external thin lines indicating the 2-LOD confidence interval. QTLs are identified by the trait code, followed by the year (2011/2012), the sowing time (A/B) and the inoculation technique (F/T) for which the QTL was detected. QTLs for FER are reported with *red bars*, FB1 in *black* and DTS in *green*. (PPTX 152 kb)
Additional file 7:
**Table S5.** List of candidate genes for *Fusarium verticillioides* resistance in the 1-LOD and 2-LOD confidence interval in the genomic regions containing QTLs for FER and FB1 contamination. (XLSX 100 kb)
Additional file 8:
**Figure S3.** Rating scale of *Fusarium* ear rot severity on the *F. verticillioides* inoculated maize ears. (PPTX 1340 kb)

## Abbreviations

ACS: Automatic cofactor selection
ACX: Acyl-CoA oxidase
AER: Aspergillus ear rot
AP2/ERF: APETALA2/ethylene responsive element binding factor
APX: Ascorbate peroxidase
DAP: Days after pollination
DEG: Differentially expressed gene
DON: Deoxynivalenol
DTS: Days to silking
ET: Ethylene
FB1: Fumonisin B1
FER: *Fusarium* ear rot
FPKM: Fragments per kilobase of transcript per million fragments mapped
GER: *Gibberella* ear rot
HSP: Heat shock protein
JA: Jasmonic acid
LG: Linkage group
MAS: Marker assisted selection
MNP: Multi nucleotide polymorphism
MQM: Multiple QTL mapping
NIRS: Near infrared spectroscopy
PDA: Potato dextrose agar
QTL: Quantitative Trait Locus
RFLP: Restriction fragment length polymorphism
RIL: Recombinant inbred line
SNP: Single nucleotide polymorphism
SSR: Single sequence repeat
ZEA: Zearalenone

## References

[1] Logrieco A, Bottalico A, Mulè G, Moretti A, Perrone G, Mulé G, Moretti A, Perrone G (2003). Epidemiology of toxigenic fungi and their associated mycotoxins for some Mediterranean crops. Eur J Plant Pathol.

[2] Santiago R, Cao A, Butrón A (2015). Genetic factors involved in fumonisin accumulation in maize kernels and their implications in maize agronomic management and breeding. Toxins (Basel).

[3] Munkvold GP (2003). Epidemiology of Fusarium diseases and their mycotoxins in maize ears. Eur J Plant Pathol.

[4] European Commission (EC). Commission Regulation (EC) No 1126/2007 of 28 September 2007 amending Regulation (EC) No 1881/2006 setting maximum levels for certainmcontaminants in foodstuffs as regards *Fusarium* toxins in maize and maize products. Off J Eur Union. 2007;L255:14–7.

[5] Rodrigues I, Naehrer K (2012). A three-year survey on the worldwide occurrence of mycotoxins in feedstuffs and feed. Toxins (Basel).

[6] Robertson-Hoyt LA, Betrán J, Payne GA, White DG, Isakeit T, Maragos CM, Molnár TL, Holland JB (2007). Relationships among resistances to Fusarium and Aspergillus ear rots and contamination by fumonisin and aflatoxin in maize. Phytopathology.

[7] Eller MS, Holland JB, Payne GA (2008). Breeding for improved resistance to fumonisin contamination in maize. Toxin Rev.

[8] Clements MJ, Maragos CM, Pataky JK, White DG (2004). Sources of resistance to fumonisin accumulation in grain and Fusarium ear and kernel rot of corn. Phytopathology.

[9] Kleinschmidt CE, Clements MJ, Maragos CM, Pataky JK, White DG (2005). Evaluation of food-grade dent corn hybrids for severity of Fusarium ear rot and fumonisin accumulation in grain. Plant Dis.

[10] Brien Henry W, Paul Williams W, Windham GL, Hawkins LK (2009). Evaluation of maize inbred lines for resistance to aspergillus and fusarium ear rot and mycotoxin accumulation. Agron J.

[11] Löffler M, Kessel B, Ouzunova M, Miedaner T (2010). Population parameters for resistance to Fusarium graminearum and Fusarium verticillioides ear rot among large sets of early, mid-late and late maturing European maize (Zea mays L.) inbred lines. Theor Appl Genet.

[12] Santiago R, Cao A, Malvar RA, Reid LM, Butrón A (2013). Assessment of corn resistance to fumonisin accumulation in a broad collection of inbred lines. F Crop Res.

[13] Lanubile A, Pasini L, Lo Pinto M, Battilani P, Prandini A, Marocco A (2011). Evaluation of broad spectrum sources of resistance to Fusarium verticillioides and advanced maize breeding lines. World Mycotoxin J.

[14] Robertson LA, Kleinschmidt CE, White DG, Payne GA (2006). Heritabilities and correlations of Fusarium Ear Rot resistance and fumonisin contamination resistance in Two maize populations. Crop Sci.

[15] Pérez-Brito D, Jeffers D, González-de-León D, Khairallah M, Cortés-Cruz M, Velasquez-Cardelas G, Aspiroz-Rivero S, Srinivasam G (2001). QTL mapping of Fusarium moniliforme ear rot resistance in highland maize, Mexico. Agrociencia.

[16] Robertson Hoyt LA, Jines MP, Balint-Kurti P, Kleinschmidt CE, White DG, Payne GA, Maragos CM, Molnar TL, Holland JB (2006). QTL mapping for Fusarium ear rot and fumonisin contamination resistance in two maize populations. Crop Sci.

[17] Ding JQ, Wang XM, Chander S, Yan JB, Li JS (2008). QTL mapping of resistance to Fusarium ear rot using a RIL population in maize. Mol Breed.

[18] Chen J, Ding J, Li H, Li Z, Sun X, Li J, Wang R, Dai X, Dong H, Song W, Chen W, Xia Z, Wu J (2012). Detection and verification of quantitative trait loci for resistance to Fusarium ear rot in maize. Mol Breed.

[19] Robertson L, Holland J, Payne G. Marker-assisted breeding for host resistance to mycotoxin contamination. In: Aflatoxin and food safety. Boca Raton: CRC Press; 2005. p. 423–36.

[20] Rafalski A (2002). Applications of single nucleotide polymorphisms in crop genetics. Curr Opin Plant Biol.

[21] Tenaillon MI, Sawkins MC, Long AD, Gaut RL, Doebley JF, Gaut BS (2001). Patterns of DNA sequence polymorphism along chromosome 1 of maize (Zea mays ssp. mays L.). Proc Natl Acad Sci U S A.

[22] Elshire RJ, Glaubitz JC, Sun Q, Poland JA, Kawamoto K, Buckler ES, Mitchell SE (2011). A robust, simple Genotyping-by-Sequencing (GBS) approach for high diversity species. PLoS One.

[23] Schnable PS, Ware D, Fulton RS, Stein JC, Wei F, Pasternak S, Liang C, Zhang J, Fulton L, Graves TA, Minx P, Reily AD, Courtney L, Kruchowski SS, Tomlinson C, Strong C, Delehaunty K, Fronick C, Courtney B, Rock SM, Belter E, Du F, Kim K, Abbott RM, Cotton M, Levy A, Marchetto P, Ochoa K, Jackson SM, Gillam B (2009). The B73 maize genome: complexity, diversity, and dynamics. Science (80-).

[24] Poland J, Endelman J, Dawson J, Rutkoski J, Wu SY, Manes Y, Dreisigacker S, Crossa J, Sanchez-Villeda H, Sorrells M, Jannink JL (2012). Genomic selection in wheat breeding using Genotyping-by-Sequencing. Plant Genome.

[25] Beyene Y, Mugo S, Semagn K, Asea G, Trevisan W, Tarekegne A, Tefera T, Gethi J, Kiula B, Gakunga J, Karaya H, Chavangi A (2013). Genetic distance among doubled haploid maize lines and their testcross performance under drought stress and non-stress conditions. Euphytica.

[26] Sonah H, Bastien M, Iquira E, Tardivel A, Légaré G, Boyle B, Normandeau É, Laroche J, Larose S, Jean M, Belzile F (2013). An improved Genotyping by Sequencing (GBS) approach offering increased versatility and efficiency of SNP discovery and genotyping. PLoS One.

[27] Spindel J, Wright M, Chen C, Cobb J, Gage J, Harrington S, Lorieux M, Ahmadi N, McCouch S (2013). Bridging the genotyping gap: using genotyping by sequencing (GBS) to add high-density SNP markers and new value to traditional bi-parental mapping and breeding populations. Theor Appl Genet.

[28] Lu F, Lipka AE, Glaubitz J, Elshire R, Cherney JH, Casler MD, Buckler ES, Costich DE (2013). Switchgrass genomic diversity, ploidy, and evolution: novel insights from a network-based SNP discovery protocol. PLoS Genet.

[29] Zila CT, Samayoa LF, Santiago R, Butron A, Holland JB (2013). A genome-wide association study reveals genes associated with Fusarium ear rot resistance in a maize core diversity panel. Genes|Genomes|Genetics.

[30] Zila CT, Ogut F, Romay MC, Gardner CA, Buckler ES, Holland JB (2014). Genome-wide association study of Fusarium ear rot disease in the U.S.A. maize inbred line collection. BMC Plant Biol.

[31] Lanubile A, Pasini L, Marocco A (2010). Differential gene expression in kernels and silks of maize lines with contrasting levels of ear rot resistance after Fusarium verticillioides infection. J Plant Physiol.

[32] Lanubile A, Bernardi J, Battilani P, Logrieco A, Marocco A (2012). Resistant and susceptible maize genotypes activate different transcriptional responses against Fusarium verticillioides. Physiol Mol Plant Pathol.

[33] Lanubile A, Bernardi J, Marocco A, Logrieco A, Paciolla C (2012). Differential activation of defense genes and enzymes in maize genotypes with contrasting levels of resistance to Fusarium verticillioides. Environ Exp Bot.

[34] Lanubile A, Ferrarini A, Maschietto V, Delledonne M, Marocco A, Bellin D (2014). Functional genomic analysis of constitutive and inducible defense responses to Fusarium verticillioides infection in maize genotypes with contrasting ear rot resistance. BMC Genomics.

[35] Lanubile A, Maschietto V, De Leonardis S, Battilani P, Paciolla C, Marocco A (2015). Defense responses to mycotoxin-producing fungi Fusarium proliferatum, F. subglutinans, and Aspergillus flavus in kernels of susceptible and resistant maize genotypes. Mol Plant-Microbe Interact.

[36] Mesterházy Á, Lemmens M, Reid LM (2012). Breeding for resistance to ear rots caused by Fusarium spp. in maize - A review. Plant Breed.

[37] Shelby RA, White DG, Bauske EM (1994). Differential fumonisin production in maize hybrids. Plant Dis.

[38] Ali ML, Taylor JH, Jie L, Sun G, William M, Kasha KJ, Reid LM, Pauls KP (2005). Molecular mapping of QTLs for resistance to Gibberella ear rot, in corn, caused by Fusarium graminearum. Genome.

[39] Martin M, Miedaner T, Dhillon BS, Ufermann U, Kessel B, Ouzunova M, Schipprack W, Melchinger AE (2011). Colocalization of QTL for gibberella ear rot resistance and low mycotoxin contamination in early European maize. Crop Sci.

[40] Martin M, Miedaner T, Schwegler DD, Kessel B, Ouzunova M, Dhillon BS, Schipprack W, Utz HF, Melchinger AE (2012). Comparative quantitative trait loci mapping for Gibberella ear rot resistance and reduced deoxynivalenol contamination across connected maize populations. Crop Sci.

[41] Munkvold GP, Desjardins AE (1997). Fumonisins in maize. Plant Dis.

[42] Reid LM, Zhu X, Parker A, Yan W (2009). Increased resistance to Ustilago zeae and Fusarium verticilliodes in maize inbred lines bred for Fusarium graminearum resistance. Euphytica.

[43] Clements MJ, Kleinschmidt CE, Maragos CM, Pataky JK, White DG (2003). Evaluation of inoculation techniques for Fusarium ear rot and fumonisin contamination of corn. Plant Dis.

[44] Bush BJ, Carson ML, Cubeta MA, Hagler WM, Payne GA (2004). Infection and fumonisin production by Fusarium verticillioides in developing maize kernels. Phytopathology.

[45] Kebede AZ, Woldemariam T, Reid LM, Harris LJ (2016). Quantitative trait loci mapping for Gibberella ear rot resistance and associated agronomic traits using genotyping-by-sequencing in maize. Theor Appl Genet.

[46] Paul C, Naidoo G, Forbes A, Mikkilineni V, White D, Rocheford T (2003). Quantitative trait loci for low aflatoxin production in two related maize populations. Theor Appl Genet.

[47] Warburton ML, Brooks TD, Windham GL, Williams WP (2011). Identification of novel QTL contributing resistance to aflatoxin accumulation in maize. Mol Breed.

[48] Busboom KN, White DG (2004). Inheritance of resistance to aflatoxin production and Aspergillus ear rot of corn from the cross of inbreds B73 and Oh516. Phytopathology.

[49] Wisser RJ, Balint-Kurti PJ, Nelson RJ (2006). The genetic architecture of disease resistance in maize: a synthesis of published studies. Phytopathology.

[50] Xiang K, Zhang ZM, Reid LM, Zhu XY, Yuan GS, Pan GT (2010). A meta-analysis of QTL associated with ear rot resistance in maize. Maydica.

[51] Holland JB. Implementation of molecular markers for quantitative traits in breeding programs - challenges and opportunities. Proc 4th Int Crop Sci Congr. 2004;1–13.

[52] Campos-Bermudez VA, Fauguel CM, Tronconi MA, Casati P, Presello DA, Andreo CS (2013). Transcriptional and metabolic changes associated to the infection by Fusarium verticillioides in maize inbreds with contrasting Ear Rot resistance. PLoS One.

[53] Acosta IF, Laparra H, Romero SP, Schmelz E, Hamberg M, Mottinger JP, Moreno MA, Dellaporta SL (2009). tasselseed1 is a lipoxygenase affecting jasmonic acid signaling in sex determination of maize. Science.

[54] Glazebrook J (2005). Contrasting mechanisms of defense against biotrophic and necrotrophic pathogens. Annu Rev Phytopathol.

[55] Christensen SA, Kolomiets MV (2011). The lipid language of plant-fungal interactions. Fungal Genet Biol.

[56] Christensen SA, Nemchenko A, Borrego E, Murray I, Sobhy IS, Bosak L, Deblasio S, Erb M, Robert CA, Vaughn KA, Herrfurth C, Tumlinson J, Feussner I, Jackson D, Turlings TCJ, Engelberth J, Nansen C, Meeley R, Kolomiets MV (2013). The maize lipoxygenase, ZmLOX10, mediates green leaf volatile, jasmonate and herbivore-induced plant volatile production for defense against insect attack. Plant J.

[57] Christensen SA, Nemchenko A, Park Y, Borrego E, Huang P, Schmelz EA, Kunze S, Feussner I, Yalpani N, Meeley R, Kolomiets MV (2014). The novel monocot-specific 9-lipoxygenase ZmLOX12 is required to mount an effective jasmonate-mediated defense against Fusarium verticillioides in maize. Mol Plant Microbe Interact.

[58] Gao X, Shim W-B, Göbel C, Kunze S, Feussner I, Meeley R, Balint-Kurti P, Kolomiets M (2007). Disruption of a maize 9-lipoxygenase results in increased resistance to fungal pathogens and reduced levels of contamination with mycotoxin fumonisin. Mol Plant Microbe Interact.

[59] Gao X, Brodhagen M, Isakeit T, Brown SH, Göbel C, Betran J, Feussner I, Keller NP, Kolomiets MV (2009). Inactivation of the lipoxygenase ZmLOX3 increases susceptibility of maize to Aspergillus spp. Mol Plant Microbe Interact.

[60] Maschietto V, Marocco A, Malachova A, Lanubile A (2015). Resistance to Fusarium verticillioides and fumonisin accumulation in maize inbred lines involves an earlier and enhanced expression of lipoxygenase (LOX) genes. J Plant Physiol.

[61] Campo S, Carrascal M, Coca M, Abián J, San Segundo B (2004). The defense response of germinating maize embryos against fungal infection: a proteomics approach. Proteomics.

[62] Pechanova O, Pechan T, Williams WP, Luthe DS (2011). Proteomic analysis of the maize rachis: potential roles of constitutive and induced proteins in resistance to Aspergillus flavus infection and aflatoxin accumulation. Proteomics.

[63] Eulgem T, Somssich IE (2007). Networks of WRKY transcription factors in defense signaling. Curr Opin Plant Biol.

[64] Rushton PJ, Somssich IE, Ringler P, Shen QJ (2010). WRKY transcription factors. Trends Plant Sci.

[65] Licausi F, Ohme-Takagi M, Perata P (2013). APETALA2/Ethylene Responsive Factor (AP2/ERF) transcription factors: mediators of stress responses and developmental programs. New Phytol.

[66] Marco F, Busó E, Carrasco P (2014). Overexpression of SAMDC1 gene in Arabidopsis thaliana increases expression of defense-related genes as well as resistance to Pseudomonas syringae and Hyaloperonospora arabidopsidis. Front Plant Sci.

[67] Tauzin AS, Giardina T (2014). Sucrose and invertases, a part of the plant defense response to the biotic stresses. Front Plant Sci.

[68] Chen L, Hou B, Lalonde S, Takanaga H, Hartung ML, Qu X, Guo W, Kim J, Underwood W, Chaudhuri B, Chermak D, Antony G, White FF, Somerville SC, Mudgett MB, Frommer WB (2010). Sugar transporters for intercellular exchange and nutrition of pathogens. Nature.

[69] Reid LM, Hamilton RI, Mather DE. Screening maize for resistance to *Gibberella* ear rot. Agric Agri-Food Can Tech Bull. 1996:196–5E.

[70] Berardo N, Pisacane V, Battilani P, Scandolara A, Pietri A, Marocco A (2005). Rapid detection of kernel rots and mycotoxins in maize by near-infrared reflectance spectroscopy. J Agric Food Chem.

[71] Michelmore RW, Paran I, Kesseli RV (1991). Identification of markers linked to disease-resistance genes by bulked segregant analysis: a rapid method to detect markers in specific genomic regions by using segregating populations. Proc Natl Acad Sci U S A.

[72] Pasini L, Stile MR, Puja E, Valsecchi R, Francia P, Carletti G, Salamini F, Marocco A (2008). The integration of mutant loci affecting maize endosperm development in a dense genetic map using an AFLP-based procedure. Mol Breed.

[73] Schuelke M (2000). An economic method for the fluorescent labeling of PCR fragments. Nat Biotechnol.

[74] Bolger AM, Lohse M, Usadel B (2014). Trimmomatic: a flexible trimmer for illumina sequence data. Bioinformatics.

[75] Li H, Durbin R (2010). Fast and accurate long-read alignment with Burrows-Wheeler transform. Bioinformatics.

[76] Li H, Handsaker B, Wysoker A, Fennell T, Ruan J, Homer N, Marth G, Abecasis G, Durbin R (2009). The Sequence Alignment/Map format and SAMtools. Bioinformatics.

[77] Garrison E, Marth G. Haplotype-based variant detection from short-read sequencing. arXiv Prepr arXiv12073907. 2012:1–9.

[78] Van Ooijen JW (2006). JoinMap® 4, Software for the calculation of genetic linkage maps in experimental populations.

[79] Van Ooijen JW (2009). MapQTL® 5, software for the mapping of quantitative trait loci in experimental populations.

[80] Voorrips RE (2002). MapChart: software for the graphical presentation of linkage maps and QTLs. J Hered.

[81] McCouch S, Cho Y, Yano M, Paul E, Blinstrub M, Morishima HKT (1997). Report on QTL nomenclature. Rice Genet Newsl.

