# Supplementary material for: QTL mapping and candidate genes for resistance to Fusarium ear rot and fumonisin contamination in maize
Source: BMC Plant Biol. 2017 Jan 21;17:20. doi: 10.1186/s12870-017-0970-1 (PMC5251214; doi:10.1186/s12870-017-0970-1)
Supplement: Additional file 1: — Figure S1. Weekly means of A) temperature, B) relative humidity and C) weekly sums of rain precipitation in years 2011 and 2012. First week corresponds to the first 7 days of flowering in the early sowing of both 2011 and 2012. Last week corresponds to the week of the harvest day for both 2011 and 2012, in the early and late sowings. (PPTX 72 kb) [file 12870_2017_970_MOESM1_ESM.pptx]

## Slide 1
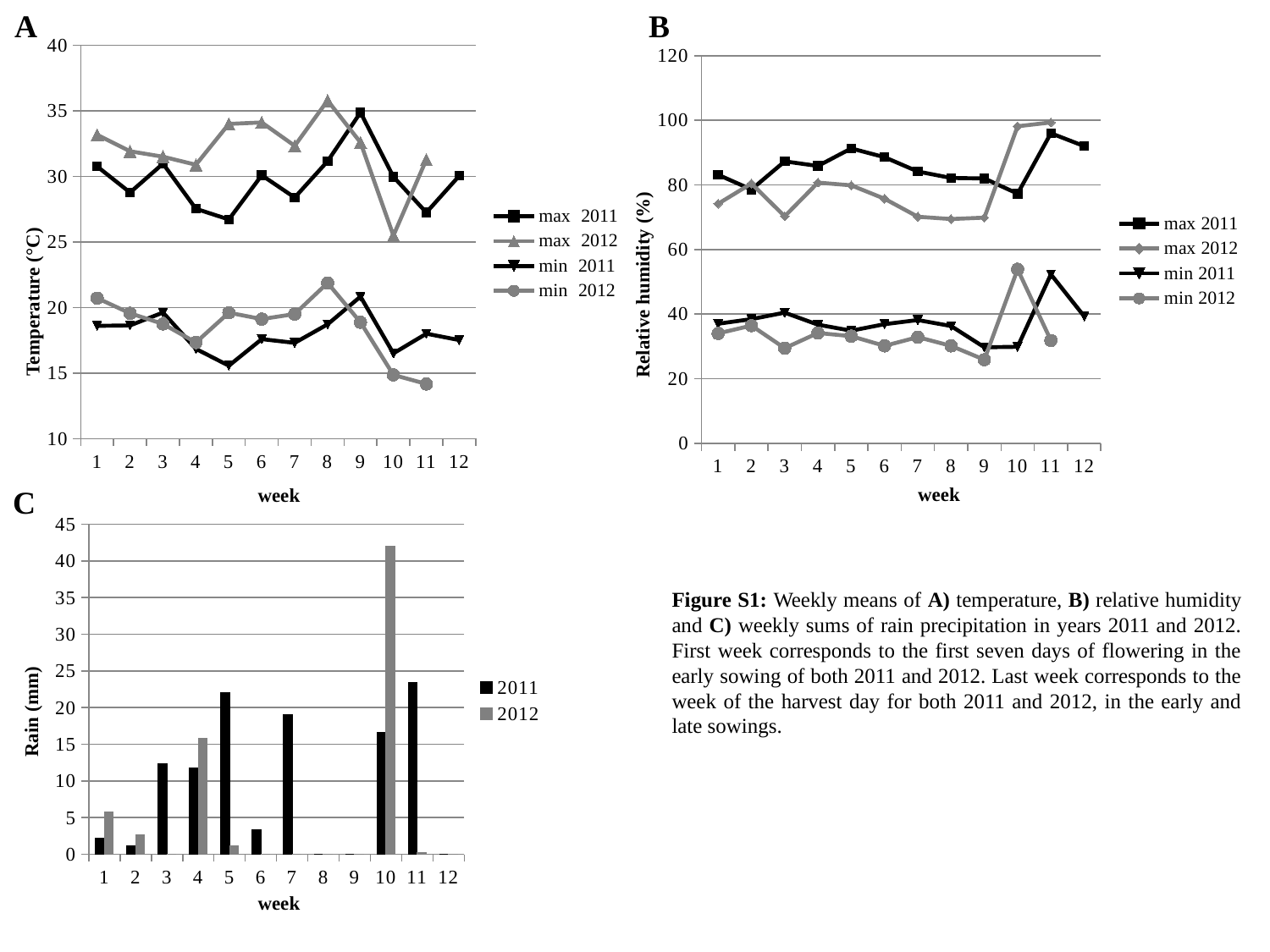

A
B
### Chart
| Category | max 2011 | max 2012 | min 2011 | min 2012 |
|---|---|---|---|---|
### Chart
| Category | max 2011 | max 2012 | min 2011 | min 2012 |
|---|---|---|---|---|Relative humidity (%)
Temperature (°C)
C
week
week
### Chart
| Category | 2011 | 2012 |
|---|---|---|Figure S1: Weekly means of A) temperature, B) relative humidity and C) weekly sums of rain precipitation in years 2011 and 2012. First week corresponds to the first seven days of flowering in the early sowing of both 2011 and 2012. Last week corresponds to the week of the harvest day for both 2011 and 2012, in the early and late sowings.
Rain (mm)
week
