# Supplementary material for: QTL mapping and candidate genes for resistance to Fusarium ear rot and fumonisin contamination in maize
Source: BMC Plant Biol. 2017 Jan 21;17:20. doi: 10.1186/s12870-017-0970-1 (PMC5251214; doi:10.1186/s12870-017-0970-1)
Supplement: Additional file 6: — Figure S2. QTLs for Fusarium ear rot (FER), fumonisin B1 contamination (FB1) and days to silking (DTS). Marker names are listed on the right side of each LG and the corresponding genetic distances (in centiMorgan) on the left side. QTLs are drawn at the right of each LG and are represented as central bars indicating the 1-LOD confidence interval and external thin lines indicating the 2-LOD confidence interval. QTLs are identified by the trait code, followed by the year (2011/2012), the sowing time (A/B) and the inoculation technique (F/T) for which the QTL was detected. QTLs for FER are reported with red bars, FB1 in black and DTS in green. (PPTX 152 kb) [file 12870_2017_970_MOESM6_ESM.pptx]

## Slide 1
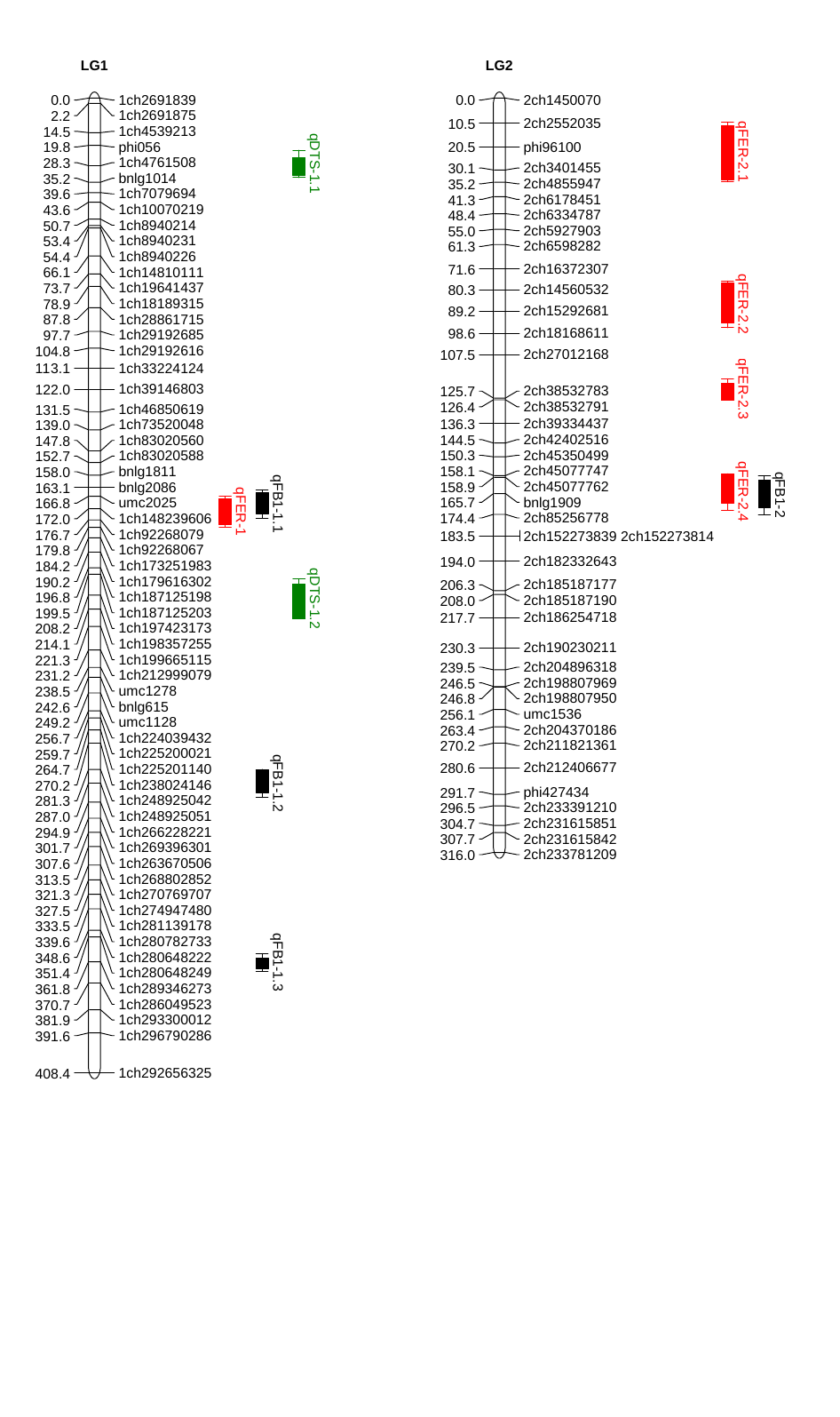

## Slide 2
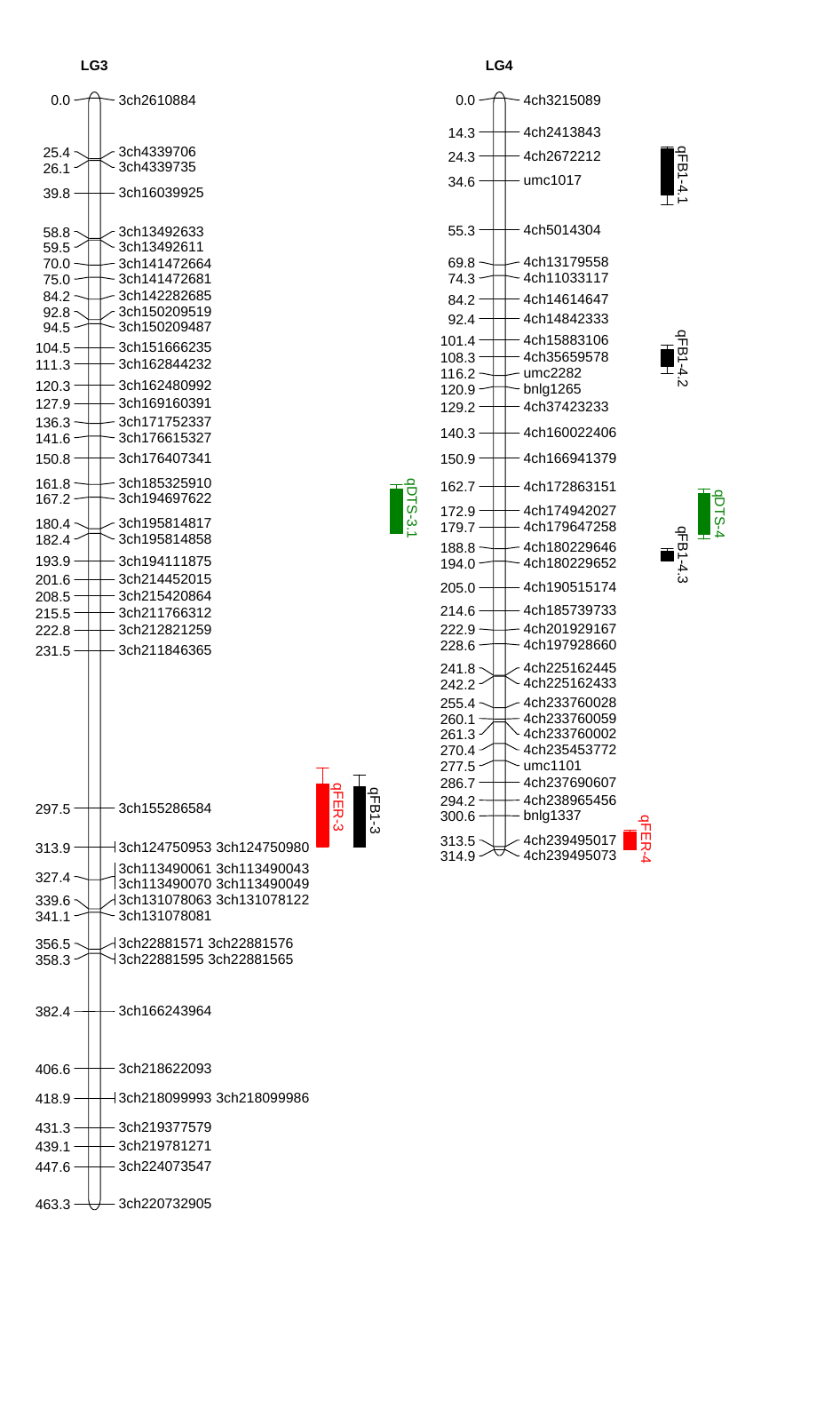

## Slide 3
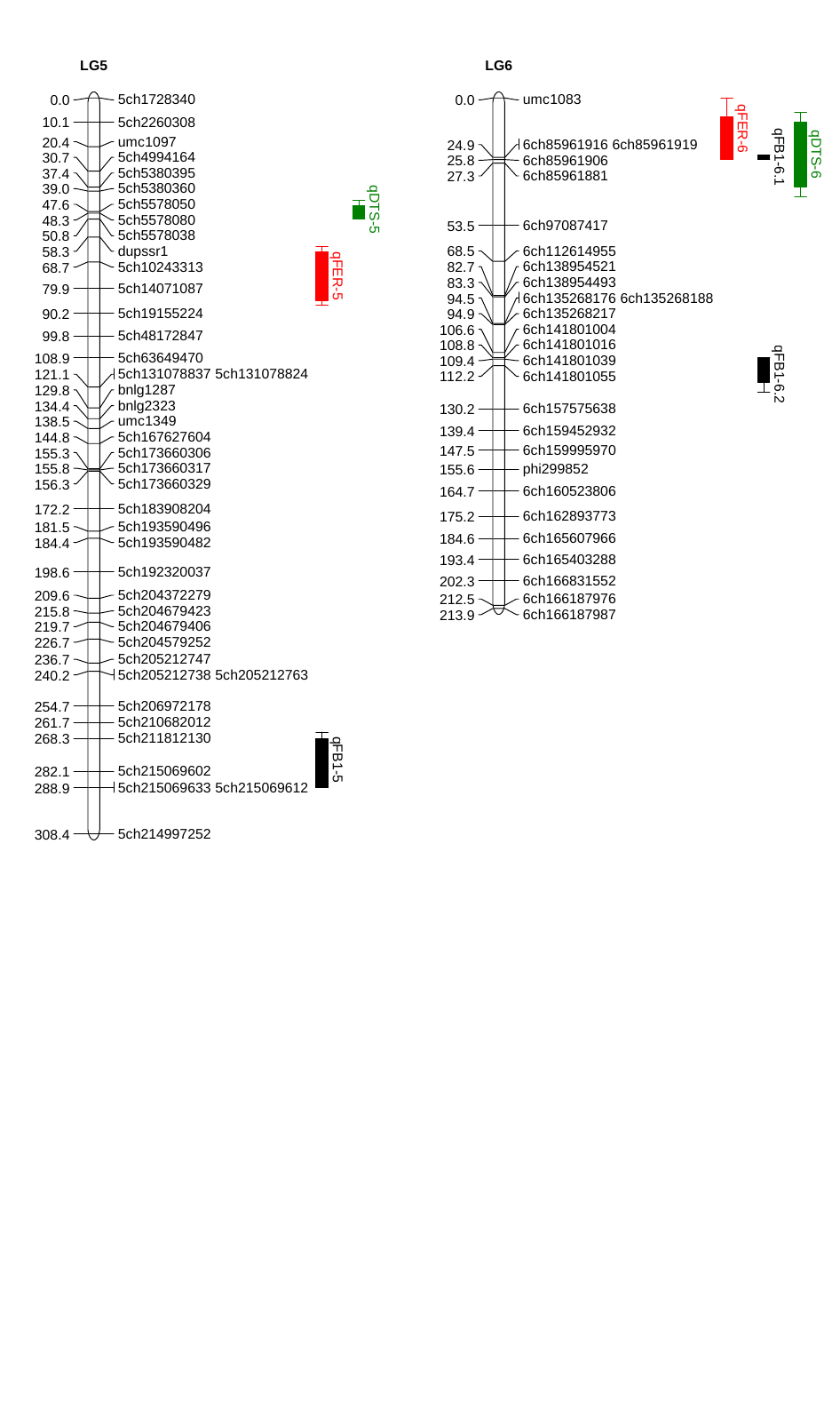

## Slide 4
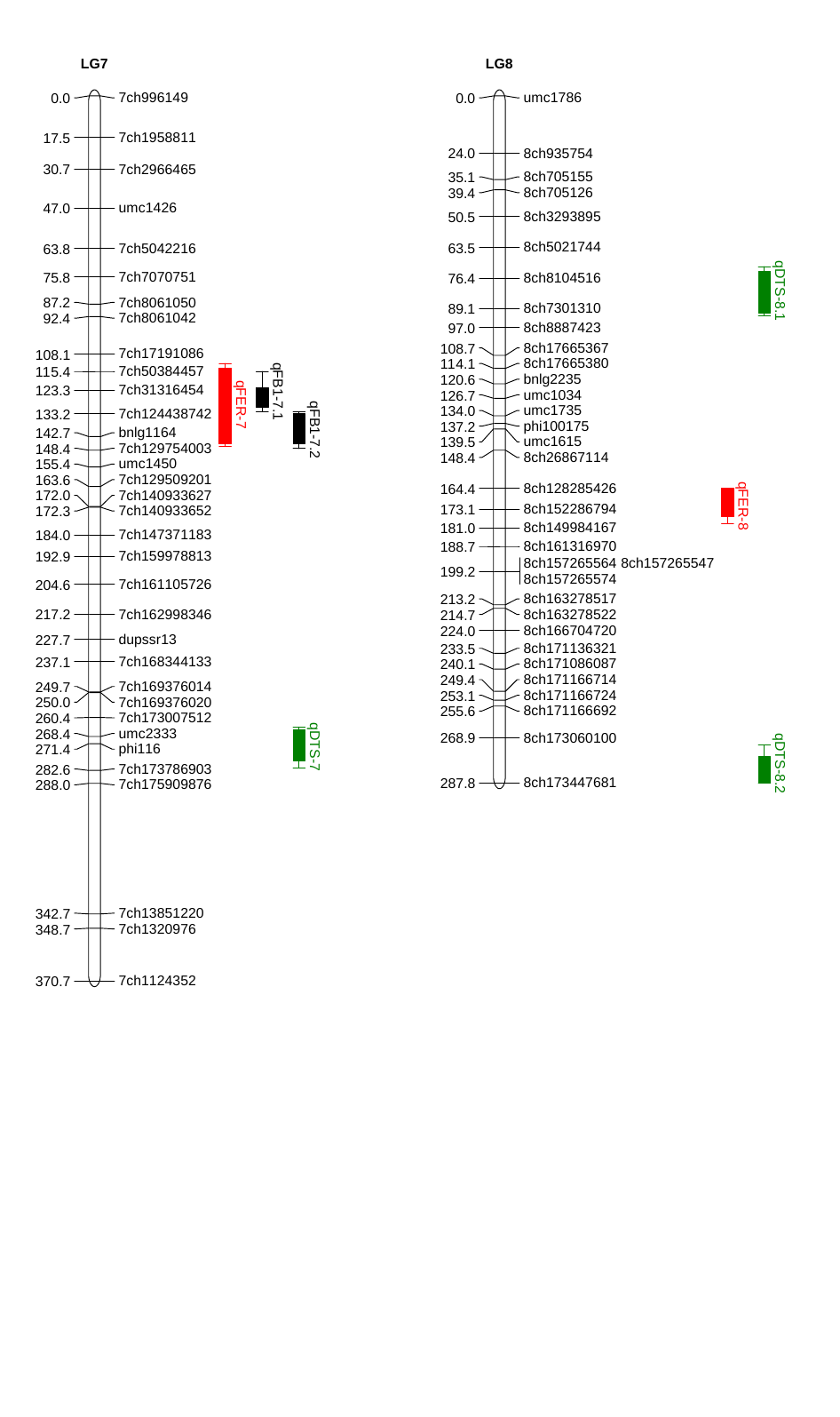

## Slide 5
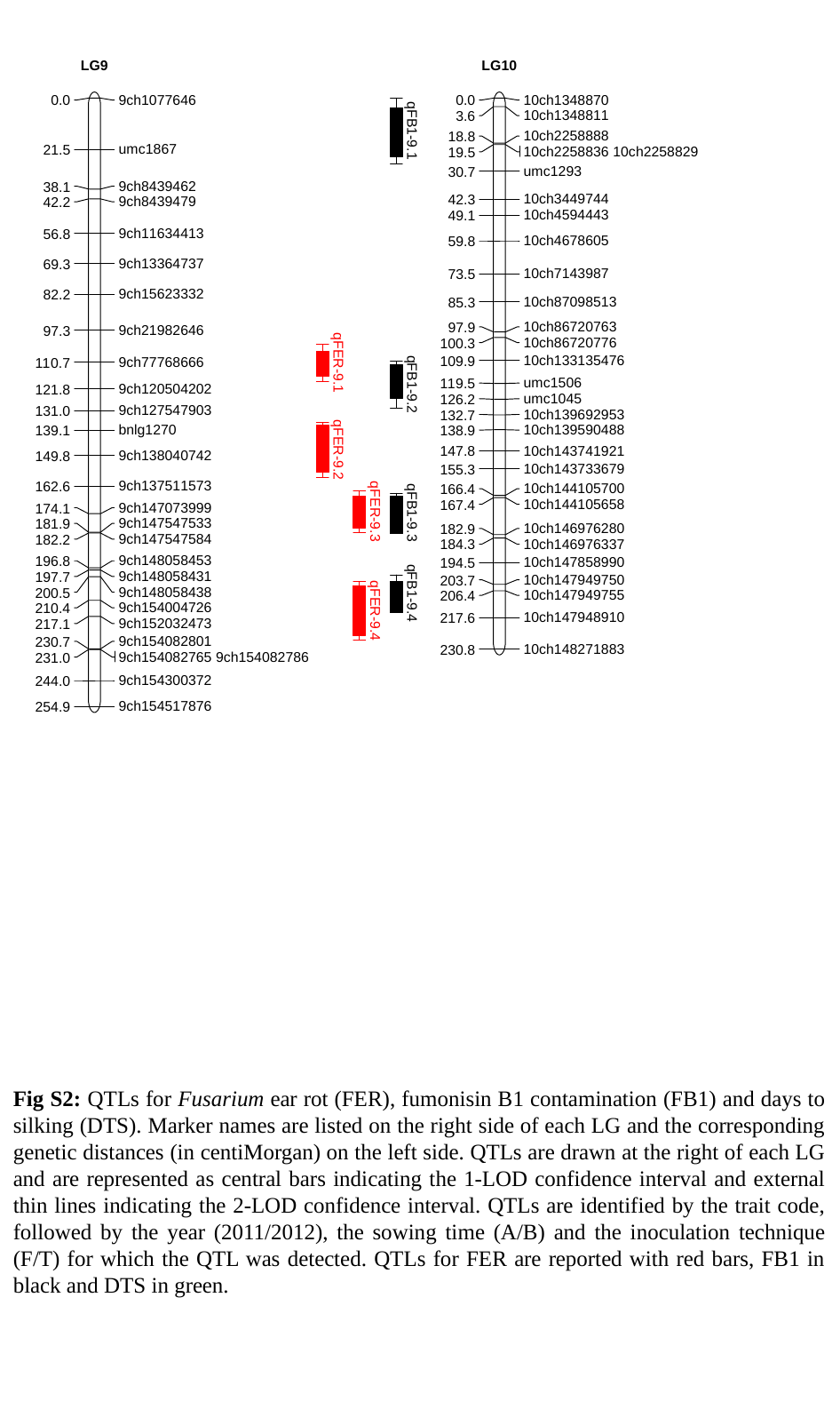

Fig S2: QTLs for Fusarium ear rot (FER), fumonisin B1 contamination (FB1) and days to silking (DTS). Marker names are listed on the right side of each LG and the corresponding genetic distances (in centiMorgan) on the left side. QTLs are drawn at the right of each LG and are represented as central bars indicating the 1-LOD confidence interval and external thin lines indicating the 2-LOD confidence interval. QTLs are identified by the trait code, followed by the year (2011/2012), the sowing time (A/B) and the inoculation technique (F/T) for which the QTL was detected. QTLs for FER are reported with red bars, FB1 in black and DTS in green.
