# Supplementary material for: QTL mapping and candidate genes for resistance to Fusarium ear rot and fumonisin contamination in maize
Source: BMC Plant Biol. 2017 Jan 21;17:20. doi: 10.1186/s12870-017-0970-1 (PMC5251214; doi:10.1186/s12870-017-0970-1)
Supplement: Additional file 8: — Figure S3. Rating scale of Fusarium ear rot severity on the F. verticillioides inoculated maize ears. (PPTX 1340 kb) [file 12870_2017_970_MOESM8_ESM.pptx]

## Slide 1
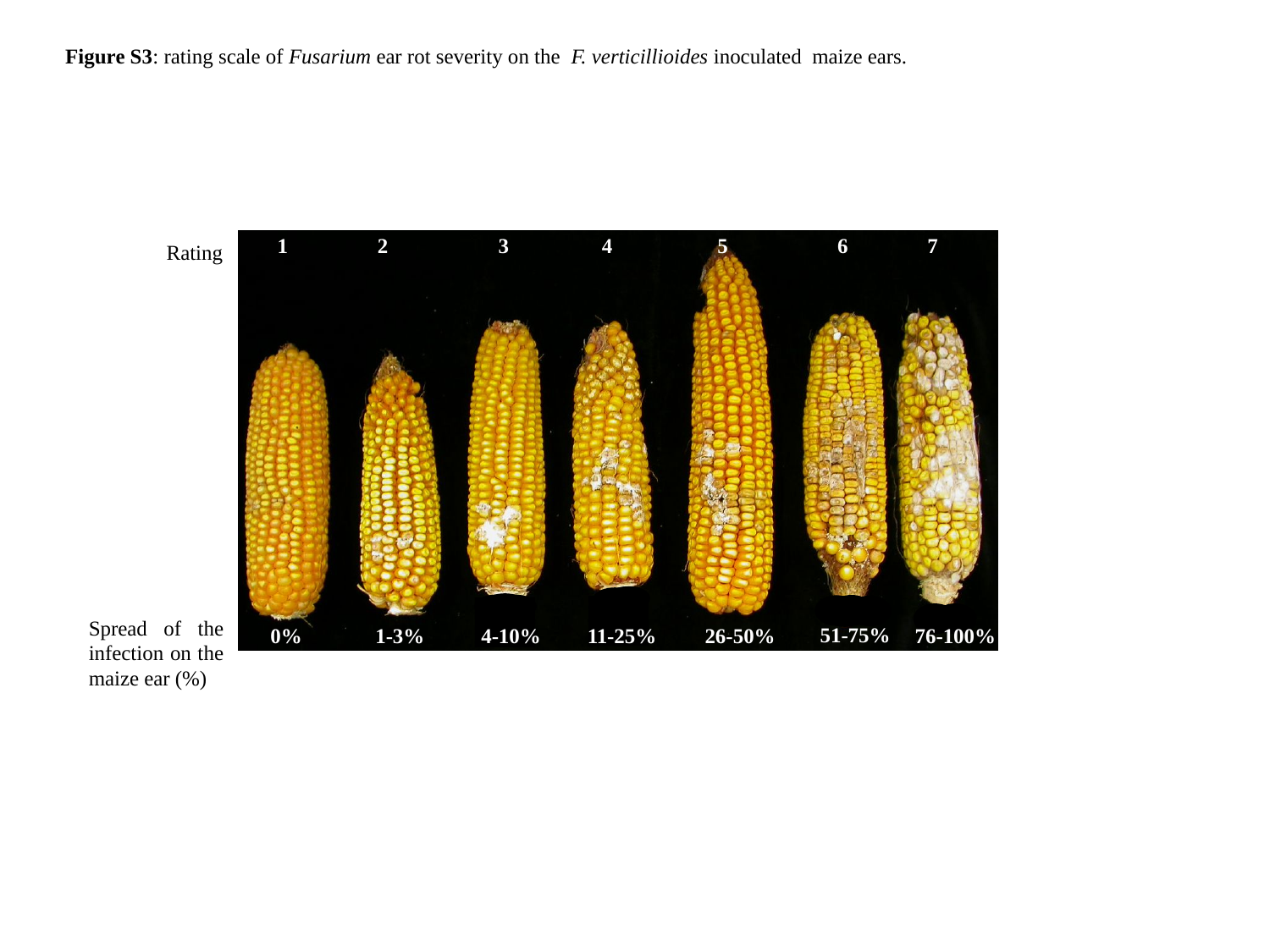

Figure S3: rating scale of Fusarium ear rot severity on the F. verticillioides inoculated maize ears.
1
2
3
4
5
6
7
Rating
Spread of the infection on the maize ear (%)
51-75%
0%
1-3%
4-10%
11-25%
26-50%
76-100%
